# Terrestrial Inputs Shape Coastal Bacterial and Archaeal Communities in a High Arctic Fjord (Isfjorden, Svalbard)

**DOI:** 10.3389/fmicb.2021.614634

**Published:** 2021-02-26

**Authors:** Lisa-Marie Delpech, Tobias R. Vonnahme, Maeve McGovern, Rolf Gradinger, Kim Præbel, Amanda E. Poste

**Affiliations:** ^1^Department of Biology, École Normale Supérieure de Lyon, Université de Lyon, Lyon, France; ^2^Department of Arctic and Marine Biology, UiT The Arctic University of Norway, Tromsø, Norway; ^3^Norwegian Institute for Water Research (NIVA), Tromsø, Norway; ^4^Norwegian College of Fishery Science, UiT The Arctic University of Norway, Tromsø, Norway

**Keywords:** Arctic, climate change, land-ocean connectivity, pelagic microbial communities, freshwater runoff, melt season, rivers and sediments, biogeochemical cycles

## Abstract

The Arctic is experiencing dramatic changes including increases in precipitation, glacial melt, and permafrost thaw, resulting in increasing freshwater runoff to coastal waters. During the melt season, terrestrial runoff delivers carbon- and nutrient-rich freshwater to Arctic coastal waters, with unknown consequences for the microbial communities that play a key role in determining the cycling and fate of terrestrial matter at the land-ocean interface. To determine the impacts of runoff on coastal microbial (bacteria and archaea) communities, we investigated changes in pelagic microbial community structure between the early (June) and late (August) melt season in 2018 in the Isfjorden system (Svalbard). Amplicon sequences of the 16S rRNA gene were generated from water column, river and sediment samples collected in Isfjorden along fjord transects from shallow river estuaries and glacier fronts to the outer fjord. Community shifts were investigated in relation to environmental gradients, and compared to river and marine sediment microbial communities. We identified strong temporal and spatial reorganizations in the structure and composition of microbial communities during the summer months in relation to environmental conditions. Microbial diversity patterns highlighted a reorganization from rich communities in June toward more even and less rich communities in August. In June, waters enriched in dissolved organic carbon (DOC) provided a niche for copiotrophic taxa including *Sulfitobacter* and *Octadecabacter*. In August, lower DOC concentrations and Atlantic water inflow coincided with a shift toward more cosmopolitan taxa usually associated with summer stratified periods (e.g., SAR11 Clade Ia), and prevalent oligotrophic marine clades (OM60, SAR92). Higher riverine inputs of dissolved inorganic nutrients and suspended particulate matter also contributed to spatial reorganizations of communities in August. Sentinel taxa of this late summer fjord environment included taxa from the class Verrucomicrobiae (*Roseibacillus*, *Luteolibacter*), potentially indicative of a higher fraction of particle-attached bacteria. This study highlights the ecological relevance of terrestrial runoff for Arctic coastal microbial communities and how its impacts on biogeochemical conditions may make these communities susceptible to climate change.

## Introduction

Arctic regions are warming two times faster than lower latitudes (Serreze and Barry, 2011; Osborne et al., 2018). Subsequent increases in the quantity (Bintanja and Selten, 2014) and variability (Bintanja et al., 2020) of precipitation, melting glaciers, and thawing permafrost are expected to lead to increased terrestrial runoff (Shiklomanov and Shiklomanov, 2003; Milner et al., 2017) alongside changes in geochemistry of the runoff (Holmes et al., 2012). Coastal environments are prominent habitats on a pan-Arctic scale with a high degree of connectivity between land and sea (McClelland et al., 2012). Coastal ecosystems can act as hotspots for carbon burial (Bianchi et al., 2018) and organic matter (OM) cycling (Bianchi et al., 2020), but are also sensitive to ongoing climate change (Jeffries et al., 2013; Bhatt et al., 2014; Fritz et al., 2017).

Aquatic microorganisms play an essential role for cycles of carbon, nitrogen, and of other elements (Arrigo, 2005; Garcia-Descalzo et al., 2013), including the recycling of OM and inorganic nutrients (Falkowski et al., 2008; Ferrera et al., 2015; Worden et al., 2015). Microbial community structure, and the degree to which their functions are realized, strongly depends on environmental conditions (Allison and Martiny, 2008; Gilbert et al., 2012; Shade et al., 2012; Louca et al., 2016). In particular, seasonal changes (e.g., in light and nutrient availability, temperature, and salinity) strongly affect microbial community function and composition (Gilbert et al., 2012; Cram et al., 2015). In coastal areas, these variables are altered by terrestrial runoff from Arctic thawing catchments (McGovern et al., 2020). In the Arctic, climate change is amplifying seasonal changes in temperature (Serreze and Barry, 2011) and terrestrial runoff (Holmes et al., 2012), and will likely affect coastal microbial communities (Kirchman et al., 2009; Vincent, 2010; Cavicchioli et al., 2019). Thus, understanding how these communities respond to environmental variables through the melt season is needed to predict how climate-influenced changes might affect microbial communities in Arctic coastal waters.

Despite the importance of microbial communities in coastal environments, their vulnerability to climate change in the Arctic, including their response to increased terrestrial runoff, is poorly understood, especially in nearshore habitats. Previous studies demonstrated a direct effect of river runoff on coastal microbial community composition and diversity (Hauptmann et al., 2016), as well as indirect effects of the physical (Han et al., 2015) and chemical conditions (Sipler et al., 2017; Müller et al., 2018; Underwood et al., 2019; Thomas et al., 2020) on seasonal (Marquardt et al., 2016; Kellogg et al., 2019) and spatial dynamics (Bourgeois et al., 2016) in microbial community composition in Arctic regions. However, very few studies have included shallow nearshore waters which are often heavily terrestrially influenced. The few available studies from Svalbard revealed spatial effects of glacial runoff on coastal marine sediment communities (Park et al., 2011; Bourgeois et al., 2016), compositional differences between seawater and sediment communities (Teske et al., 2011), and potential direct effects of glacier microbial communities on coastal communities (Garcia-Lopez et al., 2019), but remained limited to investigating one environmental compartment and one fjord, and rarely considered environmental variables (Thomas et al., 2020). In Isfjorden, Marquardt et al. (2016) highlighted a link between eukaryotic community composition and seasonally influenced variables, but with a focus on outer-fjord stations, thus reducing the potential to determine the influence of runoff-affected variables. Hence, little is known about how terrestrial runoff and underlying environmental gradients affect microbial communities in Arctic nearshore waters.

The high latitude Svalbard archipelago is of particular interest to study effects of climate change on coastal waters, as it is already characterized by higher temperatures than other Arctic systems at the same latitude due to advection of warmer ocean waters (Skogseth et al., 2020). Climate change effects are felt in Svalbard, where, due to increased precipitation and glacial melt, runoff is predicted to increase by 200% before 2100 (RCP4.5 scenario; Adakudlu et al., 2019). In Isfjorden, seasonal changes in runoff quantity and geochemistry have a pronounced impact on physico-chemical conditions in the fjord, with spring freshet in June delivering high amounts of DOC, and late season runoff in August characterized by high concentrations of dissolved inorganic nutrients (nitrate, silicate, phosphate) and terrestrial particulate matter (including particulate organic carbon, nitrogen and phosphorus) (McGovern et al., 2020).

Our microbial study focused on the Isfjorden system on the west coast of Svalbard, building on a recent study that demonstrated extensive impacts of riverine inputs on temperature, light availability, inorganic nutrients and terrestrial OM in the fjord water column (McGovern et al., 2020). We hypothesized that seasonal changes in runoff would cause seasonal and spatial variability in microbial community composition and function between the early and late summer melt season. Two main research objectives were addressed: (1) to determine how runoff affects bacterial and archaeal (thereafter referred to as microbial) community structure at the land-ocean interface and (2) to identify seasonal changes in microbial community composition and function in terrestrially influenced Arctic waters, using 16S rRNA gene sequencing from samples collected along the freshwater-marine continuum in a high Arctic fjord (Isfjorden, Svalbard). In order to target two phases in the summer melt season, the spring freshet and late-runoff, samples were collected from coastal waters in June and August. In these shallow nearshore environments, riverine inputs and high rates of sedimentation and sediment resuspension likely lead to a high degree of connectivity between coastal waters, rivers and sediments. Given these tight links between land, water and sediments, samples were also collected in several rivers feeding the fjord arms, and from sediments along the same gradients, providing important contextual data for the interpretation of our coastal water column observations. We hypothesized that terrestrial runoff would lead to spatial changes along the sampled environmental gradient, i.e., from the estuaries to the outer fjord, either directly (*via* communities transported from rivers), or indirectly (shaped by physico-chemical variables affected by runoff).

## Materials and Methods

### Sample Collection, Microbial Sample Processing, and Physico-Chemical Variable Determination

**FIGURE 1:**
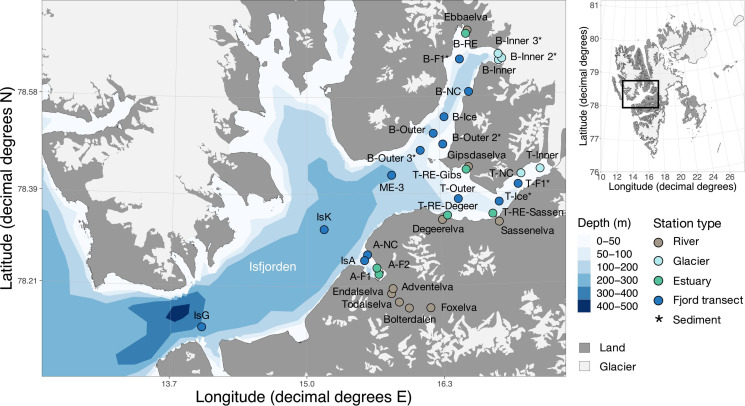
Station map of Isfjorden. Water column samples were collected from two depths in June and August 2018. Color of the symbols indicates the location in the fjord. An asterisk at the station name indicates sites where only sediments were collected. Other stations include both water column samples and sediment samples. Glaciers are represented in light-gray and land in dark-gray. The insert marks the location of the fjord in Svalbard. Details of the sample names, coordinates, location and type are available in Supplementary Table 1. The map was made using PlotSvalbard (v0.8.11) in R (Vihtakari, 2019). The Svalbard map originates from the Norwegian Polar Institute (2020, CC BY 4.0 license) and the bathymetry shapefile from the Norwegian Mapping Authority (2020, CC BY 4.0 license).

In 2018, samples were collected during two field campaigns in June and August in Isfjorden. We sampled the water column along gradients from river and glacier fronts to the outer fjord (Figure 1 and Supplementary Table 1). To assess the connectivity between water column, sediments, and rivers, we also sampled surface river water and marine sediments. As differences in the habitat characteristics of sediments, rivers and water column exceed by far seasonal differences within one habitat, we did not consider seasonality of communities for rivers or sediments. Fjord water column samples were collected from two water depths (0 and 15 m) using a Niskin bottle. Surface river water samples were collected in the main channel of eight different rivers and tributaries using a stainless-steel bucket in June and August. Surface sediment samples were collected in August using a van-Veen grab at the same marine stations as fjord water samples were collected, and subsamples of surface sediments (0-1 cm) were carefully removed from the undisturbed closed van-Veen grab. In order to avoid cross-contaminations, van-Veen grab samples were collected after completion of the water sampling using a different boat. Water and sediment samples were stored in dark insulated boxes at 4°C prior to processing at the University Center in Svalbard (UNIS) lab the same day. Subsamples for DNA analysis were filtered onto 0.2 μm pore size polycarbonate filters using a vacuum filtration system (max 200 mbar vacuum) or directly placed into 2 mL cryo vials (sediments), kept at 4°C on the boat and frozen at −20°C the same day until further processing.

Physical conditions and water chemistry were characterized in a parallel study (McGovern et al., 2020), and included in our data analysis (Table 1). Measured variables included temperature, salinity, turbidity, concentrations of dissolved and particulate organic carbon (DOC, POC), stable isotope values for POC (POC δ^13^C) and particulate nitrogen (PN δ^15^N), C:N ratio of particulate OM (POM) concentrations of chlorophyll *a*, phaeophytin, dissolved inorganic nutrients (ammonium NH_4_^+^, phosphate PO_4_^3–^, nitrite and nitrate NO_2_^–^ + NO_3_^–^, and silicate SiO_2_), and suspended particulate matter (SPM). Specific UV absorbance at 254 nm (SUVA_254_) was also measured as an indicator of aromaticity (humic acid content) of dissolved organic matter (DOM) (Weishaar et al., 2003).

**TABLE 1:** Monthly averaged (±Standard Deviation) physical and chemical properties in the water column for each water type. Standard deviations are given in parentheses. *n* gives the number of samples considered in each group for calculations. Retrieved and adapted from McGovern et al. (2020).

	**June**				**August**			
	
	**Estuary SW *n* = 10**	**Glacier SW *n* = 4**	**Fjord SW *n* = 9**	**AdW *n* = 9**	**Estuary SW *n* = 7**	**Glacier SW *n* = 3**	**Fjord SW *n* = 7**	**AdW *n* = 14**
Salinity	30.1 (7.5)	31.4 (2.7)	34.0 (0.9)	34.7 (0.3)	26.1 (11.7)	21.4 (4.1)	31.6 (2.8)	35.3 (0.5)
Temperature (°C)	3.5 (1.2)	3.6 (1.6)	3.3 (1.1)	1.8 (0.6)	6.6 (1.1)	5.3 (1.1)	6.9 (1.0)	4.5 (0.8)
Secchi depth (m)	0.7 (0.3)	1.2 (1.2)	4.8 (2.0)	3.0 (2.7)	1.7 (1.1)	1.4 (1.1)	4.0 (2.2)	3.6 (2.5)
SPM (mg.L^–1^)	29.3 (6.9)	27.0 (3.7)	26.1 (4.8)	26.0 (3.7)	74.7 (71.7)	32.6 (30.0)	25.3 (7.6)	24.2 (11.3)
DOC (μmol.L^–1^)	103.0 (18.9)	107.5 (17.1)	95.0 (7.1)	101.7 (16.0)	74.3 (15.1)	60.0 (10.0)	92.9 (16.0)	77.1 (10.7)
SUVA_254_ (m^2^.gC^–1^)	2.43 (0.53)	2.56 (0.68)	1.14 (0.80)	1.70 (0.86)	3.15 (0.78)	3.03 (0.41)	2.24 (0.43)	2.52 (0.39)
Chl *a* (mg.m^–3^)	1.31 (0.89)	0.79 (0.32)	1.54 (0.73)	1.41 (0.98)	0.92 (0.69)	1.28 (1.33)	1.06 (0.42)	0.96 (0.43)
Phaeophytin (mg.m^–3^)	0.07 (0.05)	0.05 (0.03)	0.37 (0.37)	0.09 (0.07)	0.15 (0.06)	0.11 (0.07)	0.15 (0.02)	0.13 (0.06)
PO_4_ (μmol.L^–1^)	0.64 (1.19)	0.20 (0.22)	0.64 (0.77)	0.16 (0.05)	0.22 (0.09)	0.14 (0.03)	0.17 (0.05)	0.26 (0.07)
NO_2_ + NO_3_ (μmol.L^–1^)	1.19 (1.24)	0.68 (0.26)	0.59 (0.67)	0.58 (0.25)	3.20 (4.46)	1.94 (1.08)	0.39 (0.12)	0.72 (0.45)
NH_4_ (μmol.L^–1^)	0.83 (0.38)	0.85 (0.44)	0.75 (0.57)	0.99 (0.49)	1.03 (0.40)	0.73 (0.17)	0.64 (0.13)	1.25 (0.54)
SiO_2_ (μmol.L^–1^)	4.7 (6.1)	1.9 (1.4)	1.4 (2.1)	1.0 (0.5)	10.1 (11.2)	9.1 (3.1)	3.4 (0.7)	3.5 (1.0)
POC (μmol.L^–1^)	41.0 (28.7)	17.9 (1.0)	33.8 (14.5)	24.2 (10.9)	127.9 (188.6)	23.2 (15.1)	21.9 (8.3)	19.1 (8.1)
Particulate P (μmol.L^–1^)	0.30 (0.17)	0.13 (0.02)	0.23 (0.12)	0.15 (0.09)	1.39 (2.15)	0.33 (0.25)	0.14 (0.07)	0.12 (0.09)
Particulate N (μmol.L^–1^)	4.2 (1.1)	2.7 (0.6)	4.4 (1.2)	4.1 (3.4)	10.0 (9.8)	2.9 (2.5)	3.1 (1.0)	2.9 (2.0)
POM C:N	9.8 (6.5)	6.9 (1.2)	8.1 (4.2)	6.9 (2.1)	10.0 (10.0)	9.3 (2.2)	7.0 (0.8)	7.5 (2.0)
POC δ^13^C (%o)	−28.4 (1.1)	−27.5 (0.3)	−28.5 (1.4)	−27.2 (1.3)	−27.5 (0.5)	−26.8 (0.8)	−27.0 (0.4)	−27.3 (0.9)

### DNA Extraction, Polymerase Chain Reaction, Library Preparation, and MiSeq Sequencing

Microbial genomic DNA was isolated using the DNeasy^®^ PowerSoil^®^ Kit following the kit instructions with modifications after Hassett et al. (2019). Despite its development for soil samples, the kit has been evaluated as efficient for coastal marine pelagic samples (e.g., Walden et al., 2017). Solution C1 was replaced with 600 μL Phenol:Chloroform:Isoamyl while washing with C2 and C3 was replaced with two washing steps using 850 μL chloroform. Before the last centrifugation step the column was incubated at 55°C for 5 min.

For microbial community composition analysis, we amplified the V4 region of a ca. 292 bp fragment of the 16S rRNA gene using the primers (515F, GTGCCAGCMGCCGCGGTAA and 806R, GGACTACHVGGGTWTCTAAT) (Caporaso et al., 2012; evaluated by Wear et al., 2018). The library preparation protocol is described in Wangensteen et al. (2018). The library was sequenced using the Illumina paired-end 2 × 300 bp chemistry.

### Sequence Analysis

Sequences were processed using a pipeline modified after Hassenrück (2019). Demultiplexing and primer clipping were done using cutadapt (Martin, 2011) (v2.8), and the resulting sequences were archived in the European Nucleotide Archive under project accession number PRJEB40446, run accessions ERR4653578-ERR4653672. The reads were quality trimmed with a sliding window of 4 and a quality threshold of 15 using trimmomatic (Bolger et al., 2014) (v0.39) and reads shorter than 100 bp were discarded. Remaining reads were merged using Pear (Zhang et al., 2014) (v0.9.6) with a minimum overlap of 10 bp, a minimum length of the merged reads of 200 bp (expected 292 bp), and a maximum of 500 bp. Successfully paired reads were quality controlled with FastQC (Babraham Bioinformatics, 2019) (v0.11.9), dereplicated using Swarm’s dereplication code (Mahé et al., 2015), and clustered using Swarm v2 (Mahé et al., 2015) (v3.0.0) with the fastidious option. An Operational Taxonomic Unit (OTU) contingency table was created and taxonomy of the representative amplicons assigned using SINA (Pruesse et al., 2012) (v1.6.0) against the SILVA SSU non-redundant (v138) reference database (Quast et al., 2013). Further analyses were performed in R (v3.6.3) (R Core Team, 2020), as referenced in GitHub (Delpech, 2020). Sequences related to chloroplasts or mitochondria were removed, and only OTUs with more than one sequence in more than two samples were kept, removing 9% of the sequences. A negative control was included in the sequencing, and sequences identified in this sample were removed from all samples. After these quality control steps, the average number of sequences per sample was 143,543.

Taxonomy was parsed using the curated SILVA SSU database (v138) (Quast et al., 2013, curated by Hassenrück, 2020). To further investigate taxonomic composition, remaining OTUs were pooled by taxonomic level using taxapooler v1.4 (Gobet and Ramette, 2011). Functions were predicted using Tax4Fun (Aßhauer et al., 2015), with both KEGG Ortholog (KO) and KEGG pathway reference profiles. The KEGG pathway matrix was curated and functions irrelevant to bacteria were removed. The matrix was further used as community matrix with the same methods as described in the statistical analyses. To investigate targeted functions, the KO matrix was subset to the relevant functions, using the KEGG database. Metabolic functions were determined with KO genes or enzymes related to the KEGG reaction or pathway (Supplementary Table 2).

### Statistical Analysis

To investigate temporal differences between the early and late melt-season, water column samples were grouped by month. To further investigate spatial differences within one month, fjord water samples collected from the surface and 15 m were grouped based on a temperature-salinity diagram and their sampling location. Surface waters (SW; salinity < 34.7, temperature > 1°C) were differentiated from advected waters by their salinity and temperature (AdW; salinity ≥ 34. 7, Nilsen et al., 2008). Some samples showed a mixed signal of AdW and SW (Intermediate Water) based on their salinity characteristics but were treated as SW in this study (Supplementary Figure 1). SW were further classified based on location within the fjord, leading to four water types: estuarine SW (estuary SW), glacier-front SW (glacier SW), other fjord SW (fjord SW), and AdW. River samples collected in June and August were not separated according to month and were treated as individual samples for further analysis. Marine sediments sampled along the fjord transects were not separated between locations and were analyzed in relation to the water column samples.

For statistical analysis of the data, eight samples with low sequence counts were removed, and the OTU table randomly rarefied to 42,990 sequences for further analyses. A metadata table of reads removed during bioinformatic processing and rarefaction is provided in the supplement (Supplementary Table 3). The analyses presented here were performed on the rarefied dataset; the analyses with the unrarefied dataset showed similar results. Alpha diversity estimators were calculated after repeated (*n* = 100) random subsampling of the sequences. Richness was assessed as the number of OTUs, Chao1 (Chao, 1984) and Abundance-based Coverage Estimator (ACE) (Chao and Lee, 1992) abundance-based indices (Kim et al., 2017), evenness as Pielou’s index, and diversity as Shannon’s and inverse Simpson’s indices. Rare biosphere was assessed with relative singletons (SSO) generated *de novo* after rarefaction (number of OTUs occurring only once in the sample after rarefaction). These SSOs may include artifacts, but can support the discussion about the rare biosphere (Jousset et al., 2017). To test for differences in diversity among sample groupings, Shapiro-Wilk’s test was used to test for normality, and Bartlett’s test for homoscedasticity. The *post hoc* Dunn’s test was performed on the Kruskal-Wallis test output to account for non-gaussian repartitions within some groups, and to test for pairwise differences (false discovery rate (FDR)-corrected *p-*values). To test for differences in metabolic functions, a Kruskal-Wallis test and the *post hoc* Dunn’s test were performed to account for non-parametric data with heterogeneous variances. *P-*values were adjusted using the Benjamini-Hochberg (BH) correction (Benjamini and Hochberg, 1995).

Ordinations were performed with the Vegan package in R (v2.5-6) (Oksanen et al., 2019), using relative abundances of the OTUs and rarefied data. For beta diversity, the OTU turnover between or within sites was calculated using the Jaccard index. Microbial community structure was assessed using non-metric multidimensional scaling (NMDS), based on Bray-Curtis dissimilarities, with the Wisconsin double standardization implemented in the metaMDS function. To test for differences among *a priori* sample groupings, permutational analysis of variance (PERMANOVA) (Anderson, 2017) and Analysis of Similarity (ANOSIM) (Clarke, 1993) were computed using adonis and a *post hoc* modified version (Hassenrück, 2019) of the anosim function in Vegan (FDR-corrected *p*-values). PERMANOVA provides a pseudo F-ratio and *p*-value for group-wise tests, and tests for dispersion within groups. ANOSIM tests for similarity between and among groups. ANOSIM and its *post hoc* test provide an *R*-value ranging from 0 to 1 with higher values indicating stronger differences between groups, and a FDR-corrected *p*-value for the ANOSIM *R*-value, based on 999 permutations.

To investigate the relevance of environmental variables in explaining variation in microbial community structure, principal component analysis (PCA) (based on Euclidean distances) was performed on the log-transformed environmental and Hellinger-transformed (Legendre and Gallagher, 2001) community matrices. Procrustes analysis was performed to compare ordinations, yielding a significance value. PCA and NMDS were used to explore the relationships between community data and environmental drivers by fitting variables onto unconstrained ordinations using the envfit function. Finally, redundancy analysis (RDA) was used to test hypotheses and to quantify the proportion of variance explained by the physico-chemical data. Constraining variables were selected using forward and reverse model selection with the double-stopping criterion described in Blanchet et al. (2008) implemented in the ordistep function in Vegan. Significance of each vector was tested using permutation tests (999). All constraining variables represented in the RDAs were significant (*p* < 0.05). Correlations between physico-chemical variables were tested prior to ordination using pairs, and multicollinearity of variables was tested using vif.cca (Vegan) after ordination. Other ordinations using unrarefied data, differentially abundant OTUs (ALDEx2) (Fernandes et al., 2013), Jaccard index (presence-absence) instead of Bray-Curtis, canonical correspondence analysis, and ordinations after Hellinger or centered log-ratio transformation showed similar patterns.

Indicator species analysis (Dufrene and Legendre, 1997) was used to determine taxa that significantly contributed to seasonal differences in fjord waters. The multipatt function in the indicspecies package (v1.7.9) (Cáceres and Legendre, 2009) was used to calculate Dufrêne-Legendre Indicator Value (IV), with the control set to 9999 permutations designed by the function how in the permute R package. OTUs with an IV ≥ 0.7 and a *p*-value ≤ 0.001 were considered significant indicators. Highly abundant indicators were filtered based on a 0.5% relative abundance threshold within their group. Taxonomic affiliation of highly abundant indicator taxa was further analyzed. The correlation between top seasonal indicators (chosen among indicators with relative abundance over 1%) and environmental variables was calculated using Spearman correlations to account for non-homoscedastic repartitions. Correlations and *p*-values were calculated using the rcorr function in the Hmisc package (v4.4-0) (Harrell, 2020). *P*-values were adjusted using the Benjamini-Hochberg correction with the p.adjust function in the R stats base package. All plots were generated using the ggplot2 package in R (v3.3.0) (Wickham, 2016).

## Results

### Alpha Diversity Differs Seasonally and Spatially in Isfjorden

**FIGURE 2:**
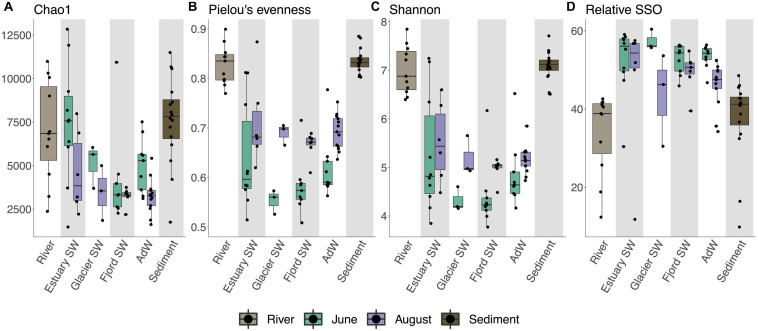
Boxplots showing alpha diversity indices according to water type and seasonal groupings. Individual data are given by black points, boxplots show the median values (line), interquartile range (box), and range of the data (whiskers). Alpha diversity indices (indicated on the left axis) were calculated as Chao1 **(A)**, Pielou’s index for evenness **(B)**, Shannon’s diversity index **(C)**, and rare biosphere as singletons (SSO) generated after rarefaction (number of OTUs occurring only once in the sample after rarefaction) **(D)**. nOTU and ACE (not shown) followed the same trend as Chao1, and inverse Simpson’s followed the same trend as Shannon’s. The bottom axis indicates the habitat (sediment, river) or water type (water column). A *post hoc* Dunn’s test was performed on the Kruskal-Wallis test output to test for differences between seasonal groups and water type groups (Supplementary Figures 2, 3 and Supplementary Table 4).

We identified a total of 35,280 Operational Taxonomic Units (OTUs) across all samples. In the water column, microbial diversity differed between June and August and between different water types. Richness was highest in June (*p* < 0.005 Chao1 and ACE), whereas evenness was highest in August (*p* < 0.001 Pielou’s) (Figures 2A,B and Supplementary Figure 2), resulting in higher Shannon’s and inverse Simpson’s diversity in August (Figure 2C and Supplementary Figure 2). However, rare taxa were more abundant in June (*p* < 0.005), but evenly distributed throughout the fjord (Figure 2D and Supplementary Figure 3). Richness decreased from the estuary SW to fjord SW and AdW (*p* < 0.05 for OTUs richness, *p* < 0.01 for Chao1 and ACE) (Figure 2A and Supplementary Figure 3). All diversity indices were significantly higher in the rivers and sediments compared to the water column, with the exception of Chao1 and ACE, which were similar between rivers and June water column, as well as between rivers and estuary SW (Figure 2A and Supplementary Figures 2, 3).

**FIGURE 3:**
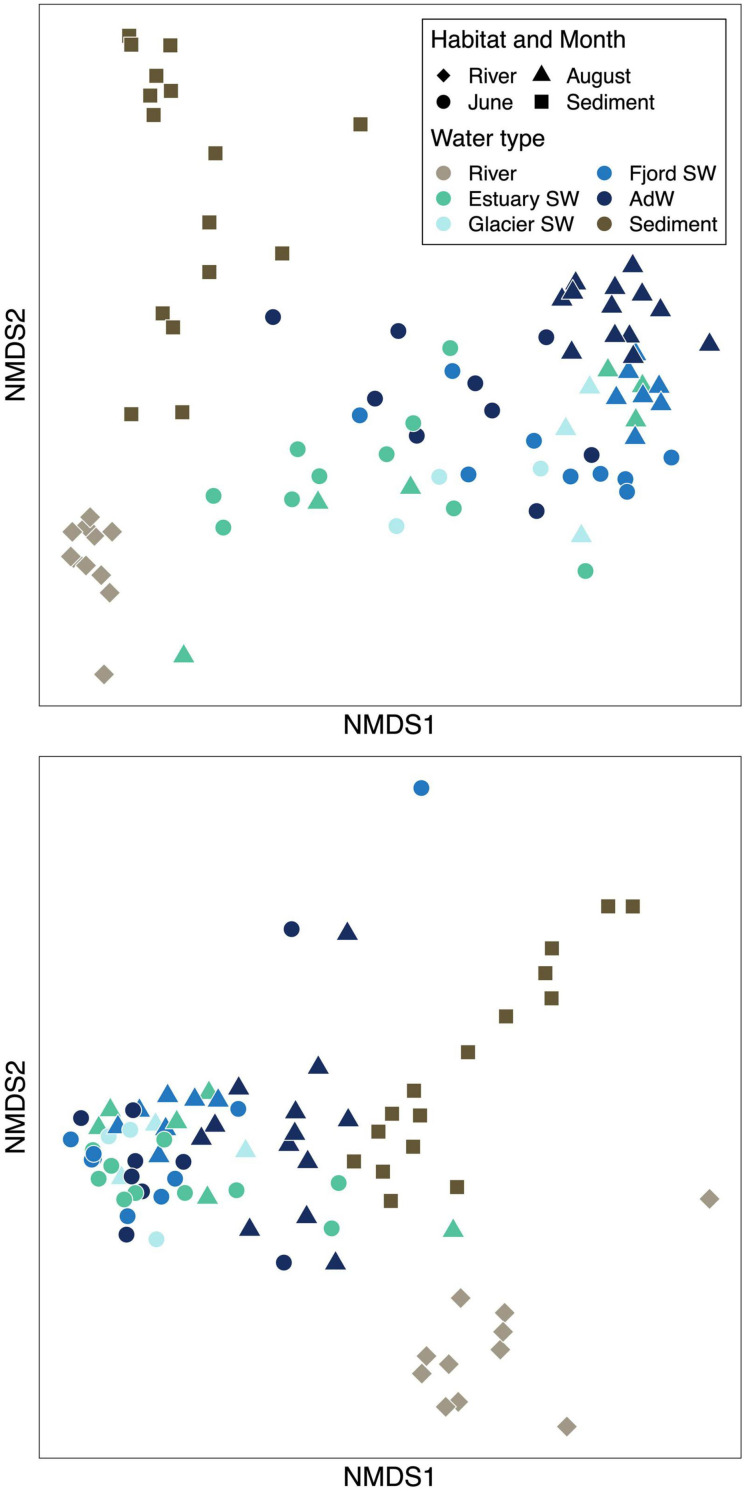
Non-metric multidimensional scaling (NMDS) ordinations showing bacterial beta diversity based on Bray-Curtis dissimilarities of **(A)** microbial community structure in the fjord (stress value = 0.15) and **(B)** functional composition based on predictions of the KEGG metabolic pathways based on OTU data (stress value = 0.06). The same legend applies to the two plots.

### Beta Diversity Reveals a Seasonal and Spatial Clustering of Microbial Communities

Microbial community structure differed seasonally (Figure 3A). In the water column, 24% of the variance in microbial community structure was explained by the sampling month (PERMANOVA, *p* = 0.001 and ANOSIM *p* < 0.001, *R* = 0.66). The OTU turnover showed that June and August water column samples shared 46% of OTUs. In August, microbial communities differed between water types (Figure 3A). In fact, 26% of the variance in microbial community structure was explained by the water type in August (PERMANOVA, *p* = 0.001). AdW significantly differed from all SW (ANOSIM, *p* < 0.01) and ANOSIM *R*-values indicated a structural gradient from estuary SW to fjord SW and AdW, with estuary SW communities being more related to river communities than any other fjord water community (*R* = 0.87 vs. 1), glacier SW more related to estuary SW than to fjord SW, and fjord SW more related to estuary SW than to AdW (*R* = 0.01 glacier SW, 0.13 fjord SW, 0.41 AdW compared to estuary SW). This spatial gradient only existed within August communities, whereas June communities were similar throughout the fjord (based on ANOSIM *R*-values and PERMANOVA).

The similarity of river and sediment communities to water column communities depended on the month and the water type (Figure 3A). June pelagic communities shared a greater proportion of OTUs with river communities (44%) than August pelagic communities (28% shared with rivers) or than sediment communities (27% shared with rivers). Based on ANOSIM *R*-values, sediment community structure was most related to estuary SW communities (*R* = 0.82), whereas differences were greater with river communities (*R* = 0.85). Finally, sediment communities (collected in August) were more similar in their structure to August water column communities (*R* = 0.90) than to June communities (*R* = 0.95), despite sharing a greater proportion of OTUs with June (37% vs. 28% with August and 27% with rivers).

Metabolic pathways predicted using Tax4Fun highlighted the same seasonal pattern as taxonomic diversity (Figure 3B). Within the water column, the sampling month explained 10% of the variance (PERMANOVA, *p* < 0.01). Functional differences between June and August were less strong than taxonomic diversity, but significant (ANOSIM *R* = 0.18, *p* < 0.001). In August, the spatial gradient detected in community structure also existed in predicted functions, and the water type explained 37% of the variance (PERMANOVA, *p* < 0.01). No spatial gradient was observed in June (PERMANOVA). The functional turnover showed a strong functional redundancy in fjord communities (96% shared functions between June and August). The sediments had a higher proportion of shared potential functions with rivers and with August samples than with June (81%, 71% and 68% resp.).

### Seasonality in Taxonomic Composition and Indicator OTUs

**FIGURE 4:**
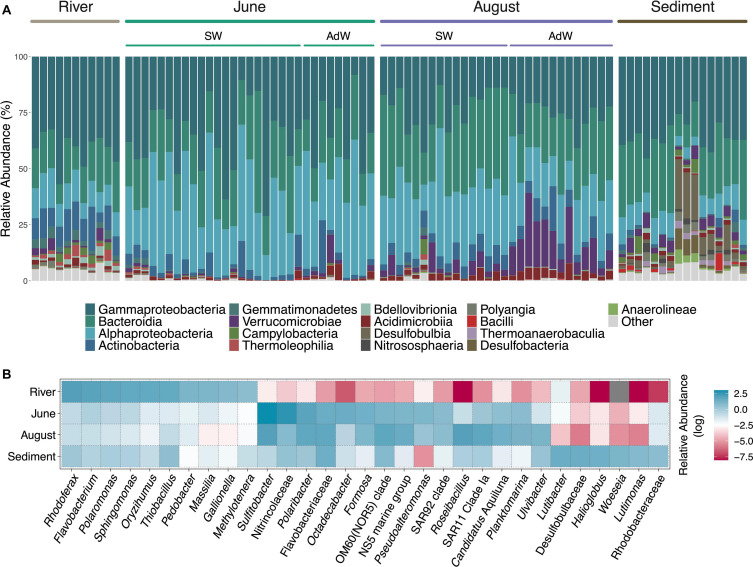
Taxonomic affiliation of abundant taxa. **(A)** Stacked barplot showing the bacterial relative abundance of the most abundant classes. For water column samples, the sampling month is indicated above the plot, and samples are further separated between surface waters (SW) and advected waters (AdW). A table summarizing relative abundances of these classes according to month and water type is provided in the Supplement (Supplementary Table 5). **(B)** Heatmap showing the mean relative abundances of the most abundant genera for each habitat and month in the water column. Relative abundances are shown on a log-scale for higher resolution. A high relative abundance is indicated by a blue color, a lower abundance is indicated by a red color, and a gray color indicates the absence of the taxon.

All communities were dominated by the phyla Proteobacteria (Alphaproteobacteria, Gammaproteobacteria) and Bacteroidetes (Bacteroidia), with different relative abundances depending on the month (Figure 4A). Fjord microbial communities in June and August were dominated by Gammaproteobacteria (36.0% and 24.2% respectively), Alphaproteobacteria (33.5% and 26.1%), and Bacteroidia (22.7% and 29.8%). In addition, clear seasonal differences existed in the abundant members of these classes (Figure 4B). Gammaproteobacteria were dominated by the order Oceanospirillales in June (17.7% of average community composition) with the family Nitrincolaceae (14.5%), and by the order Alteromonadales (4.2%) (*Pseudoalteromonas* spp.). In August, Gammaproteobacteria were dominated by Cellvibrionales (8.5%), with, e.g., oligotrophic marine clades (OM60, SAR92), while Oceanospirillales (5.4%) and Alteromonadales were less abundant (2.6%). In June, Alphaproteobacteria were dominated by the order Rhodobacterales (27.2%), highly represented by *Sulfitobacter* spp. (20.1% of community average), and *Octadecabacter* spp. (4.1%). Alphaproteobacteria were also dominated by *Sulfitobacter* spp. in August (7.7%), but other taxa were similarly abundant, including SAR11 Clade Ia (5.9%), *Planktomarina* (3.2%) and *Ascidiaceihabitans* (2.4% vs. 0.1% in June). Bacteroidia were predominantly represented by genera of the Flavobacteriales order in June, including *Polaribacter* (6.1%), *Formosa* (3.7%) and the NS5 marine group (2.8%), and by other Flavobacteriaceae (4.4%). In August, Bacteroidia also included the genus *Ulvibacter* (3.0% vs. 0.3% in June). August communities also comprised a large proportion of Verrucomicrobiae (9.1% vs. 1.1% in June), including the genera *Roseibacillus* (7.4% of average community composition), and *Luteolibacter* (1.3%). Compositional differences also existed between water types in August (Supplementary Figure 4).

River communities were dominated by Burkholderiales (33%), mostly represented by the genera *Rhodoferax* (7.9%), *Polaromonas* (5.9%), *Thiobacillus* (4.9%), *Massilia* (2.8%), and *Gallionella* (2.3%), and taxa abundant in the water column were not abundant in the rivers (Figure 4B). Sediments had a unique microbial community composition, but taxonomic overlap with both fjord water column (Flavobacteriales 19.1%, Cellvibrionales 7.4%, Rhodobacterales, Verrucomicrobiales, Oceanospirillales), and rivers (Burkholderiales, 6.8%). They showed different relative abundances in the dominant members of these groups, but some genera differentiating June from August communities were abundant in the sediments, such as OM60(NOR5) clade (2.3%), *Ulvibacter* (1.4%), *Luteolibacter* (1.2%), and *Roseibacillus* (0.8%) (Figure 4B and Supplementary Figure 4).

Indicator taxa analyses highlighted similar seasonal differences, and also pointed out several taxa of lower abundance (Supplementary Table 6). In the water column, we identified 16 indicators in June and 28 in August. We identified 35 indicators in the rivers, and 38 in the sediments. In June, these indicators included genera of the family Methylophagaceae (2.5% within June indicator taxa subset), as well as the genera *Sphingorhabdus* (3.4%), *Alcanivorax* (2.3%), *Leucothrix* (1.0%). In August, they included, for instance, members of the Actinobacteria (*Candidatus* Aquiluna (6.1% within August indicator taxa subset), *Sva0996* (2%), *Illumatobacter* (2%)), or *Fluviicola* (2.2%), *Halomonas* (2.0%), and the NS4 marine group (1.0%), and genera of the Cryomorphaceae (3.4%) and Saprospiraceae (1.4%) families. Some indicators of the sediments were redundant with the water column, mostly with August indicators, for example *Ulvibacter* (0.8% within sediment indicator taxa subset), OM60 (0.6%), Sva0996 (1.0%), and *Roseibacillus* (0.5%).

### Metabolic Functions Inferred From Taxonomy

**FIGURE 5:**
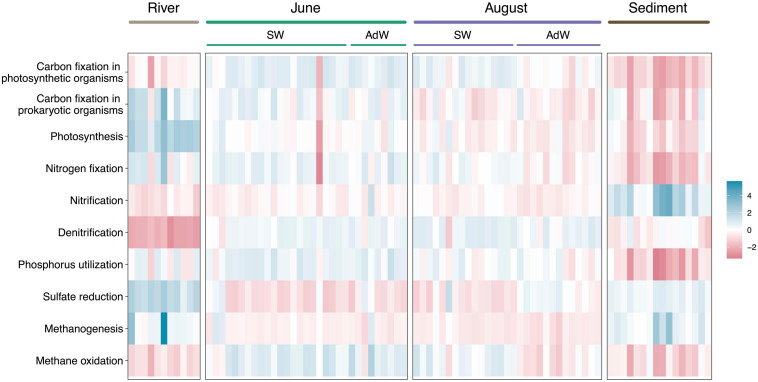
Heatmap showing differential abundances of the principal predicted functions in element cycling. Functions were predicted based on taxonomy using Tax4Fun (R). Relative abundances were z scaled to be comparable (differential abundance). For water column samples, the sampling month is indicated above the plot, and samples are further separated between surface waters (SW) and advected waters (AdW). Blue indicates a high abundance, and red a low abundance. Metabolic functions were determined with KEGG Orthologs genes or enzymes related to the KEGG reaction or pathway (Supplementary Table 2).

To investigate potential implications of taxonomic shifts on the ecosystem functioning and potential functional redundancy, we have inferred functional potentials from the taxonomy. These are predictions and cannot replace whole metagenome profiling. The relative abundance of several predicted functional potentials differed between June and August water column samples (Figure 5). June pelagic communities showed higher potential for carbon and nitrogen fixation (*p* < 0.01), phosphorus utilization (*p* < 0.005), and for methane oxidation (*p* < 0.005) compared to August pelagic communities. The latter were associated with a higher potential for sulfate reduction (*p* < 0.05) compared to June pelagic communities. In August, there was also a separation of the functional potentials between SW and AdW, highlighting the spatial gradient already observed for community taxonomic composition and structure. For instance, carbon fixation by prokaryotic organisms and methane oxidation had higher potentials in fjord SW than in AdW (*p* < 0.005), while potential for sulfate reduction was higher in AdW compared to fjord SW (*p* < 0.005) and estuary SW (*p* < 0.05).

Rivers had a high potential for photosynthesis compared to the water column (*p* < 0.001). However, river samples were segregated from water column samples by low potential for other functions, including nitrification (*p* < 0.05), denitrification (*p* < 0.001), and methane oxidation (*p* < 0.005). Finally, rivers showed high sulfate reduction potential (*p* < 0.001 compared to water column). Sediments had a specific functional footprint, with the lowest potentials for nitrogen fixation and phosphorus utilization (*p* < 0.001) and the highest potential for nitrification (*p* < 0.0001). However, sediments showed similarities with river samples in their potential for carbon fixation (in photosynthetic organisms), sulfate reduction, methane oxidation and methanogenesis (*p* > 0.05). They showed similarities with August samples in carbon fixation (in prokaryotic organisms) and photosynthesis (*p* > 0.05).

### Environmental Drivers of Pelagic Microbial Community Structure

#### Environmental Conditions in the Water Column

Table 1 summarizes the main differences in physical and chemical conditions between the two sampling months. A detailed description and discussion of environmental data is given by McGovern et al. (2020). Briefly, a freshwater footprint was observed throughout the fjord in June during the spring freshet. In August, we observed a strong gradient in salinity and turbidity, from fresh and turbid estuary water to saline and clear outer-fjord water. The physical stratification of the water column was stronger in August, when advected Atlantic water was present in sub-surface waters. Fjord water temperatures were 2 to 3°C warmer in August and generally warmest in the river-impacted estuary SW. DOC concentrations were higher in June while all major inorganic nutrients (NO_2_^–^ + NO_3_^–^, SiO_2_, particulate P and N) and POC were highest in freshwater-influenced estuary SW and glacier SW in August.

#### Environmental Drivers

**FIGURE 6:**
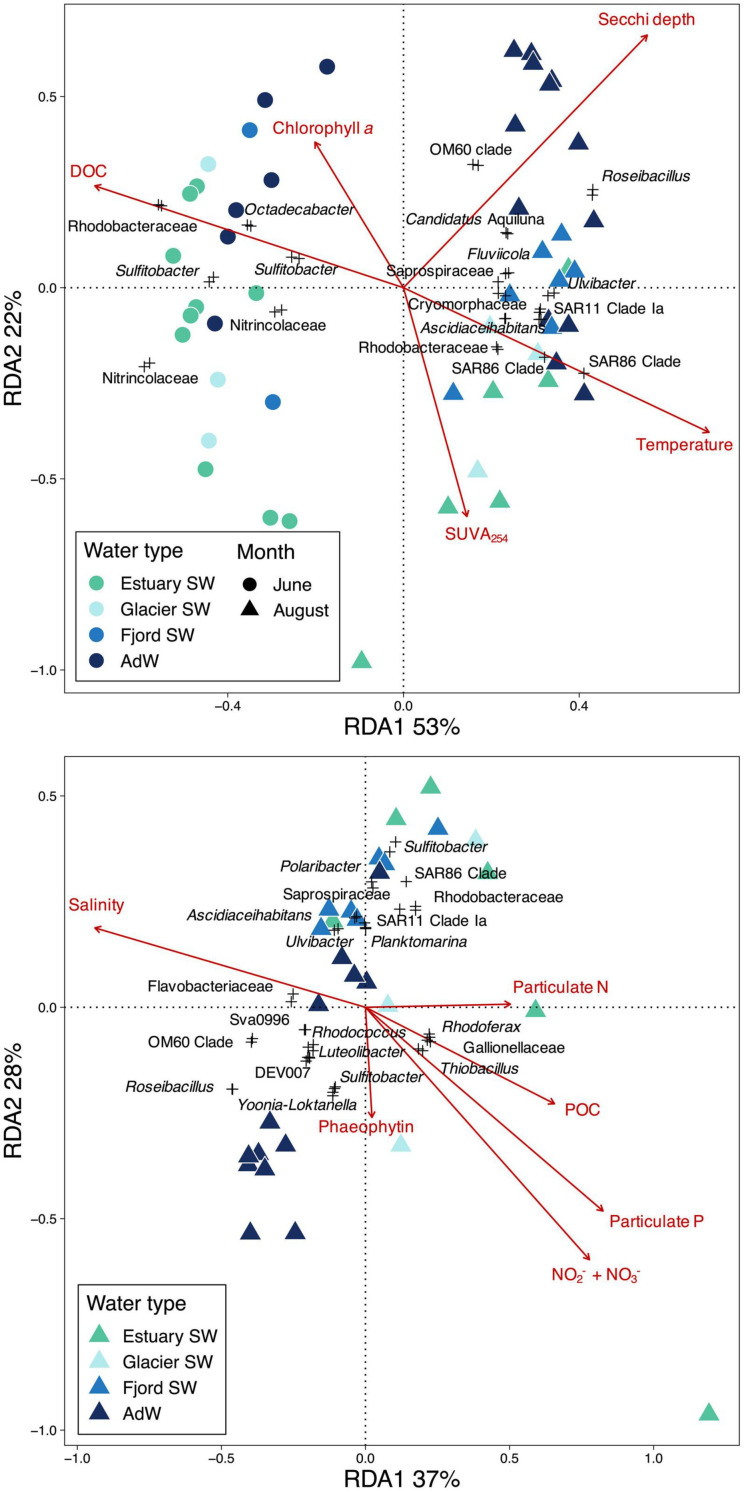
Redundancy analysis (RDA) with environmental drivers of community structure **(A)** highlighting seasonal gradients in the fjord water column, and **(B)** highlighting the spatial gradient in the fjord water column in August. Significant constraining variables are indicated in red. RDAs were run on OTUs, and only OTUs with an RDA score > 0.07 are represented and are scaled by 3. The name of the closest related genus or family (highest specified taxonomic resolution) is given for these OTUs. Percentages indicate the amount of variance explained by each axis.

Microbial community structure was significantly correlated with coastal environmental conditions in Isfjorden (Procrustes, *p* = 0.001). The observed changes in microbial community structure coincided with seasonal and spatial changes in physical and chemical conditions influenced by terrestrial inputs. Results of RDA highlighted the importance of temperature, Secchi depth, and SUVA_254_, as well as DOC and chlorophyll *a* in explaining seasonal changes between June and August communities (Figure 6A). While June community structure correlated with higher DOC and chlorophyll *a* concentrations, August community structure was related to higher temperature and SUVA_254_, especially in the estuary SW. The two RDA axes explained 75% of the total variance in community structure, with 53% of the total variance explained by the first RDA axis, which was related to the seasonal change in community structure. The shift in community structure from June to August also coincided with higher values of δ^13^C of POC (Table 1). Results of redundancy analysis on functional data also highlighted this seasonal shift (Supplementary Figure 5). June community functional structure was associated with higher DOC concentrations, while August community functional structure was associated with higher salinity and δ^13^C of POC, suggesting that the functional seasonality was mostly explained by changes in terrestrial inputs and DOC availability. Temperature did not explain this functional separation, in contrast to the taxonomic community structure ordination.

Separation along the second RDA axis followed the spatial gradient in August community structure (Figure 6A). RDA on August communities highlighted the importance of physical and chemical variables for explaining spatial variations along the fjord gradient (Figure 6B). Both RDA axes explained the spatial gradient in August, and were related to salinity, NO_2_^–^ + NO_3_^–^, particulate P, particulate N, and POC, suggesting that changes in community structure along the fjord coincided with gradients in salinity, nitrogen and particulate nutrients. 65% of the variance within August communities was explained by these variables. The inclusion of river samples in the RDA (Supplementary Figure 6) showed that salinity, turbidity, DOC, and SiO_2_, explained the transition from rivers to estuary SW. These variables explained 90% of the variance within the whole river-fjord system communities, of which 70% explained the separation between river and water column communities (first axis).

**FIGURE 7:**
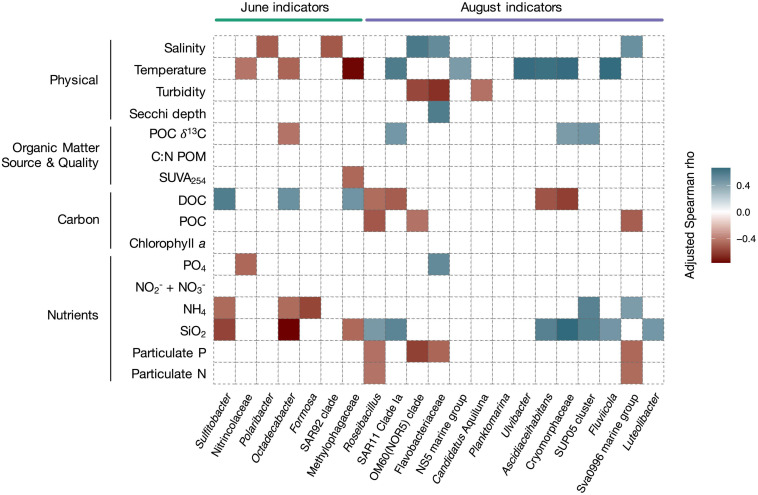
Heatmap showing significant Spearman correlations (adjusted Spearman rho) between relative abundance of seasonal indicator taxa and Isfjorden physico-chemical variables. The indicators for the two months were statistically retrieved using the indicspecies package in R (IV ≥ 0.7, *p* ≤ 0.001). A regression was done between the relative abundance of the indicator taxa and the environmental variables using individual data. The sampling month is indicated above the plot and the taxonomic affiliations are indicated beneath the plot. Relevant indicators were chosen among highly abundant indicators and are ordered by decreasing abundance for each month. For a full overview of indicator species, see Supplementary Table 6. Only significant correlations are shown. A blue color indicates a positive correlation and a red color indicates a negative correlation. *p*-values were FDR-corrected with the BH correction.

Correlations between highly abundant indicators and physico-chemical variables corroborated these results (Figure 7). The relative abundance of June indicator taxa correlated with conditions representing marine and terrestrial sources of carbon (positive correlations with DOC concentration, negative correlations with δ^13^C of POC and SUVA_254_). Correlations were strongest for highly abundant and specific indicators (e.g., *Sulfitobacter*, *Octadecabacter*; Figure 7). June indicators also correlated negatively with temperature. August indicator taxa correlated with conditions representing late melt season (positive correlation with temperature), and were negatively correlated to carbon indicators (DOC, POC). They also correlated positively with values of δ^13^C of POC. Numerous August indicators positively correlated with silicate and ammonia concentrations, nutrients which were associated with riverine inputs (McGovern et al., 2020).

## Discussion

### Terrestrial Inputs Shape Changes in Pelagic Microbial Community Structure

Our study revealed temporal and spatial shifts in microbial community structure and alpha diversity in Isfjorden. These changes were correlated with various physical and geochemical gradients driven, in part, by terrestrial inputs. Stratification and temperature of the water column were the two main physical drivers. In August, increased freshwater retention at the surface and a deeper influx of AdW led to a strongly stratified water column (McGovern et al., 2020). Meanwhile, microbial communities were significantly different between SW and AdW. As physical boundaries, like the halocline, can limit the dispersion of microbial communities (Galand et al., 2009; Treusch et al., 2009; Comeau et al., 2012; Han et al., 2015), we suggest that the stratification of the water column occurring in August may shape the observed differences in alpha and beta diversity between June and August. Water column temperature was a main explanatory variable of the temporal changes in community structure. Higher fjord water temperatures in August reflect seasonal increases in air temperature from June to August, but also the effects of river inputs. Besides warmer river waters in August, the associated high particle concentration (high SPM nearshore) led to an increased absorption of solar radiation, locally increasing temperatures in the rivers and freshwater impacted parts of the fjord (McGovern et al., 2020). Temperature may have important direct effects on shaping microbial community structure (e.g., Gilbert et al., 2009; Kirchman et al., 2009). However, we suggest that other correlating variables, such as decreased solar radiation (Alonso-Sáez et al., 2006) or changes in runoff quantity and quality (e.g., inorganic nutrients, DOC) may be equally important (Kirchman et al., 2005, 2009). Although temperature appeared to be an important explanatory variable for temporal changes in taxonomic community structure, it was unrelated to temporal changes in the predicted functional community structure, suggesting different drivers of taxonomic and functional communities (discussed below).

**FIGURE 8:**
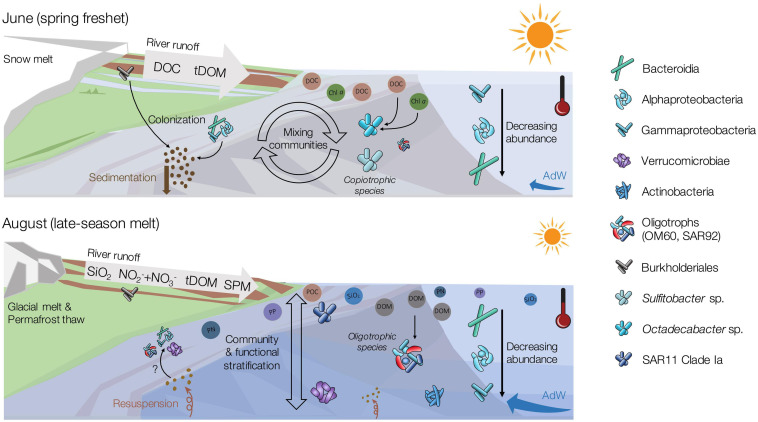
Conceptual figure depicting major seasonal changes in the microbial communities in relation to changing physical and chemical variables between the early (June) and late (August) melt season. This figure includes components of conceptual figures presented by Kellogg et al. (2019); McGovern et al. (2020), and some symbols are modified after the Integration and Application Network (ian.umces.edu/symbols/). For detailed information on biogeochemistry of the system, see McGovern et al. (2020).

Geochemical variables correlated with the temporal shift in community structure included DOC, chlorophyll *a* and SUVA_254_. In June, runoff from snowmelt on land delivered large amounts of DOC to the fjord (McGovern et al., 2020). Coinciding low values of δ^13^C in June support a mainly terrestrial source of organic carbon. Nevertheless, chlorophyll *a* concentrations were slightly higher in June, when remnants of the spring phytoplankton bloom were still present in the water column (McGovern et al., 2020). Given the post-phytoplankton bloom conditions and higher δ^13^C values in AdW, marine DOC derived from phytoplankton likely represented an additional, autochthonous source of DOC in June. Phytoplankton blooms are known to trigger bacterial succession (e.g., Teeling et al., 2012; Chafee et al., 2018; Jain et al., 2020; Manna et al., 2020). In fact, we did observe several taxa attributed to spring-bloom successions (e.g., *Polaribacter*, discussed below). The utilization of both marine and terrestrial carbon sources over the summer months could explain why DOC concentrations were lower, and DOM potentially more refractory in August (higher SUVA_254_, Weishaar et al., 2003; Spencer et al., 2012) (McGovern et al., 2020). Moreover, glacial and permafrost thaw-fed freshwater inputs in August were likely richer in humic acids than in June (higher SUVA_254_, McGovern et al., 2020) (Fellman et al., 2010; Holmes et al., 2012; Feng et al., 2013), suggesting a shift in DOM quality. Overall, the temporal shifts in community structure from June to August occurred alongside changes in DOC origin (snowmelt, spring bloom, and permafrost thaw) and quantity (high runoff in June), driven both by the spring phytoplankton bloom, and by the changes in terrestrial runoff (Figure 8). In contrast to our coastal study, seasonal microbial successions in open ocean systems are typically solely explained by changes in temperature, stratification, solar irradiance, and depletion in macronutrients (phosphorus, combined nitrogen) (Treusch et al., 2009). The importance of DOC in our study suggests that, in the nearshore, temporal shifts are also strongly influenced by the physical and geochemical effects related to terrestrial runoff.

In August, the spatial gradient in SW from estuaries to the fjord, but also between SW and AdW coincided with increased NO_2_^–^ + NO_3_^–^, particulate P, particulate N, and POC concentrations nearshore. These variables were also strongly influenced by river runoff in this system (McGovern et al., 2020). The increased stratification at this time likely facilitated the formation of these spatial gradients. As terrestrial particulate matter and inorganic nutrients have potentially strong implications for microbial community structure (Gilbert et al., 2012; Jain et al., 2019), this suggests that the runoff may also have played a role in shaping spatial changes in community structure in August. Overall, our study supports the fact that seasonality (reflected by temperature and spring bloom characteristics) is a strong driver of community structure (e.g., Gilbert et al., 2009; Kellogg et al., 2019). However, in addition, the existence of spatial changes in community structure along environmental gradients demonstrated the importance of other variables affected by the runoff (stratification, availability of organic and inorganic dissolved and particulate substrates) in shaping nearshore coastal microbial communities.

### Temporal Reorganizations of Dominant Taxa in Response to Environmental Changes

A temporal shift in dominant taxa occurred alongside changes in geochemical conditions. In June, high amounts of terrestrial and autochthonous DOC provided a niche for copiotrophic bacteria. Alphaproteobacteria were then dominated by *Sulfitobacter* spp., a chemoorganotrophic aerobic clade (Sorokin, 1995) reported in highly productive environments, mostly following phytoplankton blooms (Lee et al., 2019), and *Octadecabacter* spp., a typical polar marine species (Gosink et al., 1997). *Octadecabacter* is psychrophilic and heterotrophic (Gosink et al., 1997), but lacks the machinery needed to degrade aromatics (Newton et al., 2010), which is likely why it was less abundant in August, when the DOM shifted to a higher aromaticity (Figure 8). The genus *Polaribacter* was abundant both in June and August. Its abundance in June was not surprising since *Polaribacter* sp. is known to be a copiotrophic species that thrives in environments with high concentrations of phytoplankton exudates (Teeling et al., 2012; Xing et al., 2015). The similar abundance of *Polaribacter* sp. in both months corresponds to its ability to utilize a wide spectrum of OM, including phytoplankton derived OM (Xing et al., 2015) as well as other sources of DOM (Sipler et al., 2017; Underwood et al., 2019).

SAR11 Clade Ia is mostly found in oligotrophic systems. Hence, its higher abundance in August compared to June underlines the shift to an oligotrophic system (Rappé and Giovannoni, 2003; Malmstrom et al., 2007; Straza et al., 2010). SAR11 is typically abundant at the later stages of bacterial successions following phytoplankton spring blooms (Treusch et al., 2009), when nutrient concentrations are typically low and surface waters often stratified (Field et al., 1997; Morris et al., 2002; Bakenhus et al., 2018; Lee et al., 2019). However, we found SAR11 Clade Ia to correlate positively with silicate, temperature and marine POM, suggesting that SAR11 could be benefiting from stratified oligotrophic waters but also river inputs. Overall, SAR11 may not be only represented by oligotrophic strains in our study, as some ecotypes potentially thrived in less oligotrophic environments. Recent findings by Delmont et al. (2019) showed an environmentally mediated selection of SAR11 ecotypes. In fact, SAR11 consists of numerous subgroups that cannot all be related to specific environmental variables (e.g., Carlson et al., 2009; Eiler et al., 2009). However, this hypothesis cannot be resolved with OTU resolution, and further in-depth metagenomic analyses may be needed. Alongside the reduction in DOC in August, a population of Cellvibrionales outnumbered Oceanospirillales and Alteromonadales which were abundant in June, mostly with oligotrophic marine clades (OM60, SAR92). The abundant presence of oligotrophic marine clades in coastal waters with low chlorophyll *a* concentrations has also been observed in terrestrially influenced Beaufort sea lagoons (Kellogg et al., 2019). These observations could suggest that terrestrially influenced and warmer waters along Arctic coastlines may allow the domination of cosmopolitan taxa at the end of the melt season, potentially explaining the observed higher evenness in August (Figure 8).

Taxa of the class Verrucomicrobiae thrived in August. The two most abundant genera of this class, *Roseibacillus* and *Luteolibacter*, have also been observed in other Svalbard fjords (Cardman et al., 2014; Jain et al., 2019) and are known to colonize POM because of their ability to degrade complex carbohydrates (Jain et al., 2019; Zorz et al., 2019). Our observation supports previous findings that these bacterial groups are linked to the availability of complex organic substrates (Jain et al., 2019) in late summer. This is potentially related to the availability of polysaccharides (Cardman et al., 2014) generated by the phytoplankton bloom, and to the load of SPM from runoff (Figure 8). However, in our study, *Roseibacillus* was negatively correlated with POC concentrations, while *Luteolibacter* was not correlated with POC at all. This contrast to earlier studies might be related to the changing origin of the POM between June (marine, snowmelt) and August (permafrost-thaw, glacier melt) (McGovern et al., 2020; Venturini et al., 2020), which were not investigated separately. Hence, the known bacterial temporal successions occurring during summer stratified periods (Carlson et al., 2009; Treusch et al., 2009) also appeared influenced by river runoff and subsequent import of DOM and POM, favoring different microbial communities.

### Taxonomic Reorganizations Have Potential Implications for Coastal Ecosystem Functioning

While functional inferences from taxonomy can be misleading and cannot replace whole metagenome profiling (Aßhauer et al., 2015), careful consideration of functional characteristics can be useful for predicting potential implications of taxonomic shifts for ecosystem functioning and resilience, and can thus help inform future studies (e.g., Laroche et al., 2018). In contrast to the low taxonomic redundancy (46%), a strong functional redundancy existed between June and August communities (96%), indicating a functionally resilient ecosystem (Walker, 1992). The relative abundance of taxa capable of chemo- or phototrophic inorganic carbon fixation decreased from June to August, suggesting an increased role of heterotrophic bacteria following the spring phytoplankton bloom, which had occurred in early May 2018 (McGovern et al., 2020). The higher abundance of microbial taxa with the potential for N_2_ fixation in June could be indicative of allochthonous inputs of diazotrophs through rivers from Arctic terrestrial systems (Kim et al., 2008; Komárek et al., 2012; Vonnahme et al., 2016). However, cyanobacterial abundance was low in both months in the water column (relative abundance < 0.5%), suggesting that non-cyanobacterial diazotrophs could be important in coastal systems. Low inorganic nitrogen levels can enhance N_2_ fixation, while the high DOC concentrations can support heterotrophic diazotrophs (Zehr and Capone, 2020). The importance and ecological controls of heterotrophic diazotrophs is still an understudied topic (Zehr and Capone, 2020). However, our findings are consistent with previous associations of heterotrophic diazotrophs with high DOC, particles, or algae (Farnelid et al., 2019), indicating that they may be important during the spring freshet in the coastal Arctic.

Potential sulfur-oxidizers (SOB) and anaerobic sulfate-reducers (SRB) occurred in all investigated habitats. SRB (e.g., *Thiobacillus* spp.) were most abundant in rivers, but surprisingly also relatively abundant in deeper fjord water samples in August, potentially due to sediment resuspension (discussed below). The presence of potentially anaerobic bacteria may also reflect a change in hydrology and permafrost active layer depth, with increased contributions from groundwater and deeper soil layers in August, in contrast to snow melt and surface soils in June. However, it should be noted that presence does not necessarily imply activity. For example, bacteria potentially capable of other functions related to anoxic environments, such as methane oxidation, were more abundant in the brackish surface layer in August. While not all bacteria were necessarily active, they could be useful as biomarkers for terrestrial inputs.

### Allochthonous Taxa in the Water Column

Our results indicate that water column communities were not isolated, but supplemented by taxa introduced from rivers, resuspended sediments and advected Atlantic waters into the dynamic Isfjorden system. While terrestrial inputs appeared to have strong indirect effects on water column microbial communities through impacts on biogeochemical conditions, their role as a source of abundant taxa to fjord waters was minor. The observed temporal changes in the composition of the abundant biosphere were independent from the transport of allochthonous freshwater-taxa from the rivers. We identified taxa of the Verrucomicrobiae class as potential sentinels for August river-influenced waters, which could indicate that they are freshwater-taxa entering the fjord from rivers. However, previous studies found that direct transport from terrestrial runoff is not the main source for their abundance in marine coastal environments, but that temperature, water column depth, and nitrogen concentration are factors that influence taxonomic composition of Verrucomicrobiae (Freitas et al., 2012). Other August indicator taxa, including SAR11 Clade Ia, *Ulvibacter*, *Ascidiaceihabitans*, or *Yoonia-Loktanella*, were likely not supplied by the rivers either, as they were very rare in river samples. This supports our hypothesis that the compositional shift in August was mostly caused by alterations of the physico-chemical environment rather than to the direct transport of freshwater-taxa.

While rivers were not a source of abundant taxa to the fjord, they were likely a source of rare taxa. Their impact on community structure appeared through temporal and spatial changes in diversity indices, especially those weighing the rare biosphere (SSO, richness). The lower OTU richness and higher OTU evenness observed in August compared to June is consistent with observations in the Beaufort sea lagoons (Kellogg et al., 2019), which are also strongly impacted by river inputs in summer. We suggest that the high load of freshwater that enters the fjord in June with the spring freshet (McGovern et al., 2020) can temporally boost the richness, through addition of allochthonous taxa from the rivers into the rare biosphere. This is supported by the greater proportion of OTUs shared between rivers and June waters than with August, and more generally by the demonstrated presence of river taxa in estuaries (Fortunato et al., 2013; Hauptmann et al., 2016). Increased richness in estuaries in both months also indicates that river inputs directly supplied the rare biosphere, but the survival of those bacteria in the fjord remains unknown. We hypothesize that these taxa did not survive until August (lower richness), making them ecologically negligible compared to the stronger reorganization in the abundant biosphere, but this cannot be conclusively resolved as singletons may include artifacts or dead cells.

Linkages also existed between sediment microbial communities and both river and fjord water column communities. Similarities between sediment and river communities were likely due to the particle-attached bacteria that enter the fjord *via* runoff and settle out. This theory is supported by the observation that June pelagic communities, more influenced by river taxa, were more connected with sediments compared to August, based on the amount of shared OTUs and the higher richness. However, sediment communities also shared structural characteristics and taxa with pelagic communities, with estuarine microbial communities closer to sediments in their structure than river communities. These structural connections suggest that particles (SPM) associated with river runoff are colonized by unique microbial assemblages with overlap from both river and fjord communities, that later constitute sediment communities (Figure 8). Indeed, particles can act as a direct substrate for bacterial utilization and harbor particle-attached microbial communities with specific structure and function (Jain et al., 2019). Interestingly, we observed a different connectivity based on functional diversity, with sediments more related to rivers and August than to June pelagic community functional structure. This could be explained by sediment resuspension transporting sediment taxa back up into the water column in the estuaries. In addition, some indicators of August water column communities, such as *Ulvibacter*, *Yoonia-Loktanella*, *Illumatobacter*, and *Planktomarina*, were found enriched in the particle-attached fraction in Kongsfjorden (Jain and Krishnan, 2017), pointing to potential particle-specialized taxa. We also found some indicators in August (SAR11 Clade Ia, *Ulvibacter*, *Roseibacillus* and *Luteolibacter*) that were relatively abundant in the sediments, but not in the rivers. These observations suggest a higher fraction of particle-attached bacteria in August. These two findings, the connectivity with sediment resuspension and the convergence toward potential particle-attached bacteria, could explain the higher proportion of shared functions between sediments and August water column.

A third potential source of taxa could be through the advection of Atlantic waters. The association of several indicator taxa with AdW in August stratified waters, including *Luteolibacter*, *Roseibacillus* or the OM60 clade, indicates that advection could be an important source of taxa in August. However, considering the different repartition of these taxa in the water column, their origin remains unclear and their repartition is likely an intertwining of different sources and of different responses to the environmental conditions in the water column.

### Outlook and Perspectives

We interpreted the observed microbial community reorganization between the early and late melt season in relation to physical and biogeochemical drivers. However, other potentially relevant explanatory variables, i.e., protist or metazoan grazing, virus lysis, or bacteria-bacteria relationships, were not considered in this study. Whereas the relative importance of top-down and bottom-up effects on bacterial communities is still discussed, bottom-up effects can be prevalent in post-bloom conditions in the Arctic, when bacterioplankton communities are stimulated by resource supplied by the phytoplankton bloom (Vaqué et al., 2008). Overall, the strong seasonality in environmental conditions in the Arctic may favor bottom-up regulation of microbial communities (Gilbert et al., 2012).

Our study highlighted the high degree of connectivity between rivers, coastal waters and sediments. The presence of both autochthonous and allochthonous taxa was detected within the water column, but a selection for or against them through seasonally and spatially changing environmental conditions (Figure 8). Coastal environments are dynamic systems where terrestrial runoff and advection of marine waters converge and contribute to the formation of microbial communities. The influence of inputs from land on environmental conditions in these coastal waters further contributed to shaping the observed microbial community assemblages.

As one of the first studies in an Arctic fjord system with this spatial resolution, considering two timepoints in the melt season, our study is an important step in understanding possible future effects of increased terrestrial inputs on microbial communities along Arctic coastlines. Amplicon sequencing provided an in-depth overview of the prokaryotic variants in our system and identified potential sentinels for the observed changes in the water column biogeochemistry between the early and late melt season. However, it is not deprived of biases, from the extraction protocol, the PCR biases engendered by the primers for certain taxa (Wear et al., 2018), the gene copy number, and informatics processing. The abundances provide relative knowledge but absolute quantification with, e.g., CARD-FISH (Kubota, 2013) would be needed to compare across other studies. The possibility to include eDNA from dead cells is another bias that could be addressed using metatranscriptomics. Although amplicon sequencing is unreliable for powerful functional analyses, which require targeting key genes using metagenomics or quantitative PCR, it could aid in developing further hypotheses on expected changes in element cycling. To test for taxonomic and functional differences, an integrated “-omics” study would be needed, combined with activity measurements. Here, amplicon sequencing is useful to hypothesize possible temporal changes, but does not identify sentinels of temporal changes and terrestrial inputs, or exact perturbations of the biogeochemical cycles. Bioassay experimentations using an integrated approach with amplicon sequencing and DOM characterization with FT-ICR MS (Sipler et al., 2017) would facilitate characterization of specific changes in response to DOM availability and molecular composition. Finally, because of short-term variability in the oceanographic conditions in stratified Arctic fjords (Skarðhamar and Svendsen, 2010), the design of this study only provides a “snap-shot” of Isfjorden’s microbial communities in summer stratified months. However, it does serve as an important baseline for future studies, which should include high spatial, seasonal and interannual resolution for predicting future changes, as well as benthic community analyses and eukaryotic microbial community analyses, to provide an in-depth overview of the system.

## Data Availability Statement

The sequence data generated for this study can be found in the online ENA European Nucleotide Archive under project number PRJEB40446. The environmental data are published by McGovern et al. (2020). The processed OTU and Taxonomy tables are openly available in the UiT data archive Dataverse (https://doi.org/10.18710/JDWLVA). The scripts generated and used for this study are available on GitHub at (https://github.com/lmdelpech/16S).

## Author Contributions

AP and MM took the samples in the field and developed the sampling design. L-MD, TV, AP, and KP initiated and designed the 16S barcoding study. L-MD did the lab work and the bioinformatic analyses with support from TV and KP, did the statistical analyses with support from MM and TV, and wrote the manuscript with contributions from all co-authors. All authors contributed to the article and approved the submitted version.

## Conflict of Interest

The authors declare that the research was conducted in the absence of any commercial or financial relationships that could be construed as a potential conflict of interest.
