# Supplementary material for: Terrestrial Inputs Shape Coastal Bacterial and Archaeal Communities in a High Arctic Fjord (Isfjorden, Svalbard)
Source: Front Microbiol. 2021 Feb 26;12:614634. doi: 10.3389/fmicb.2021.614634 (PMC7952621; doi:10.3389/fmicb.2021.614634)
Supplement: Supplementary file 1 [file Data_Sheet_1.PDF]

Supplementary Material

1 Supplementary Figures

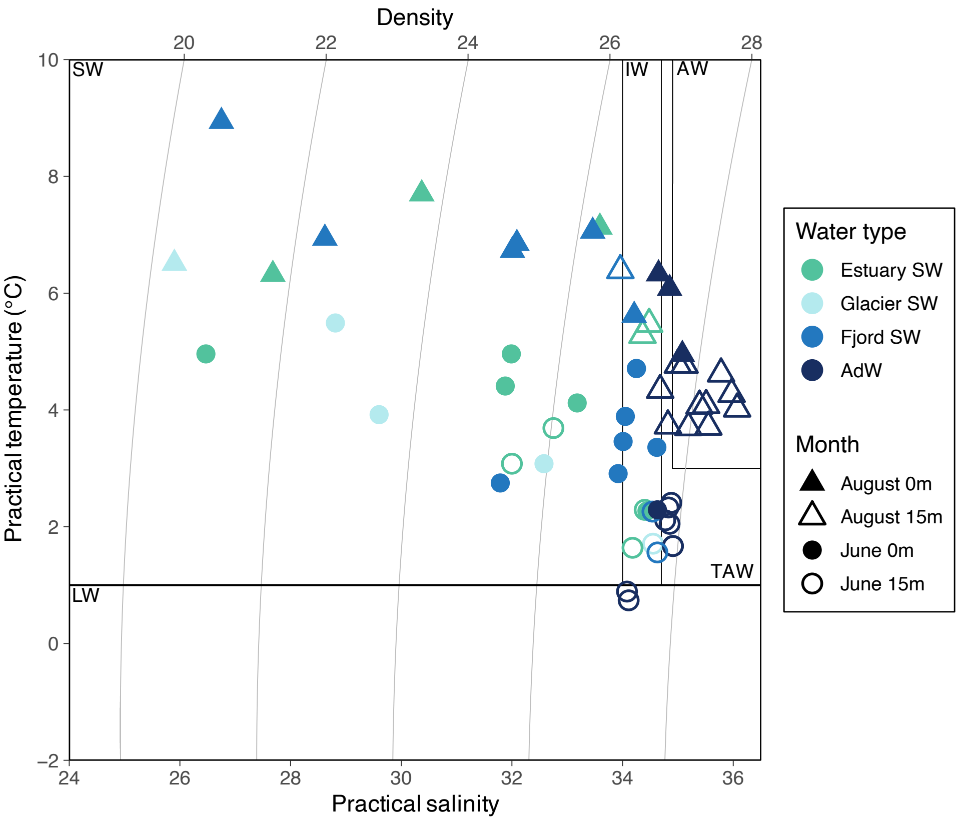

Supplementary Figure S1 | Temperature-Salinity diagram for Isfjorden defining sample classification into water types. The points show the classification of the samples taken at the surface (0m, plain symbol) and at 15m (empty symbols) in June and August into the four defined water types: Estuary Surface Water (Estuary SW), Glacier SW, Fjord SW, and Advected Waters (AdW). Water masses abbreviations: SW: Surface Water, IW: Intermediate Water, AW: Atlantic Water, TAW: Transformed Atlantic Water, LW: Local Water. The diagram was made using the *ts_plot* function from the R package PlotSvalbard (Vihtakari, 2019) and water masses for Isfjorden (Nilsen et al., 2008).
