# Supplementary material for: Terrestrial Inputs Shape Coastal Bacterial and Archaeal Communities in a High Arctic Fjord (Isfjorden, Svalbard)
Source: Front Microbiol. 2021 Feb 26;12:614634. doi: 10.3389/fmicb.2021.614634 (PMC7952621; doi:10.3389/fmicb.2021.614634)
Supplement: Supplementary file 3 [file Data_Sheet_3.PDF]

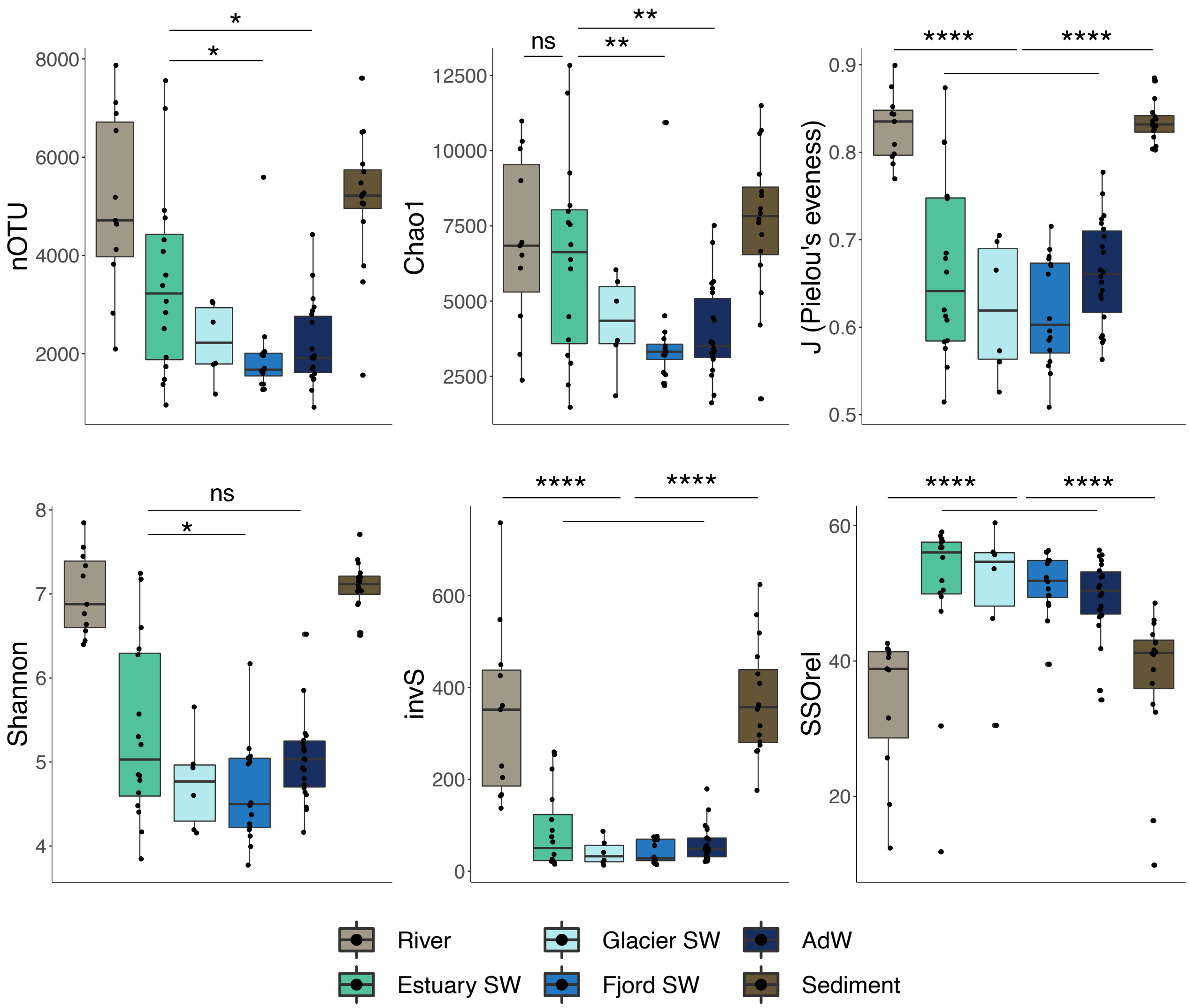

Supplementary Figure S3 | Boxplots showing alpha diversity indices for water type groupings. Individual data are given by black points, boxplots show the median values (line), interquartile range (box), and range of the data (whiskers). Alpha diversity indices were calculated as number of OTUs, Chao1 and ACE abundance-based richness, Shannon and inverse Simpson diversity indices, Pielou's index for evenness, and rare biosphere as singletons (SSO) (number of OTUs occurring only once in the sample after rarefaction). ACE (not shown) followed the same trend as Chao1. Posthoc Dunn's test was performed on Kruskal-Wallis tests: **** $p \leq 0.0001$, *** $p \leq 0.001$, ** $p \leq 0.01$, * $p \leq 0.05$ (FDR-corrected *p*-values).
