# Supplementary material for: Terrestrial Inputs Shape Coastal Bacterial and Archaeal Communities in a High Arctic Fjord (Isfjorden, Svalbard)
Source: Front Microbiol. 2021 Feb 26;12:614634. doi: 10.3389/fmicb.2021.614634 (PMC7952621; doi:10.3389/fmicb.2021.614634)
Supplement: Supplementary file 4 [file Data_Sheet_4.PDF]

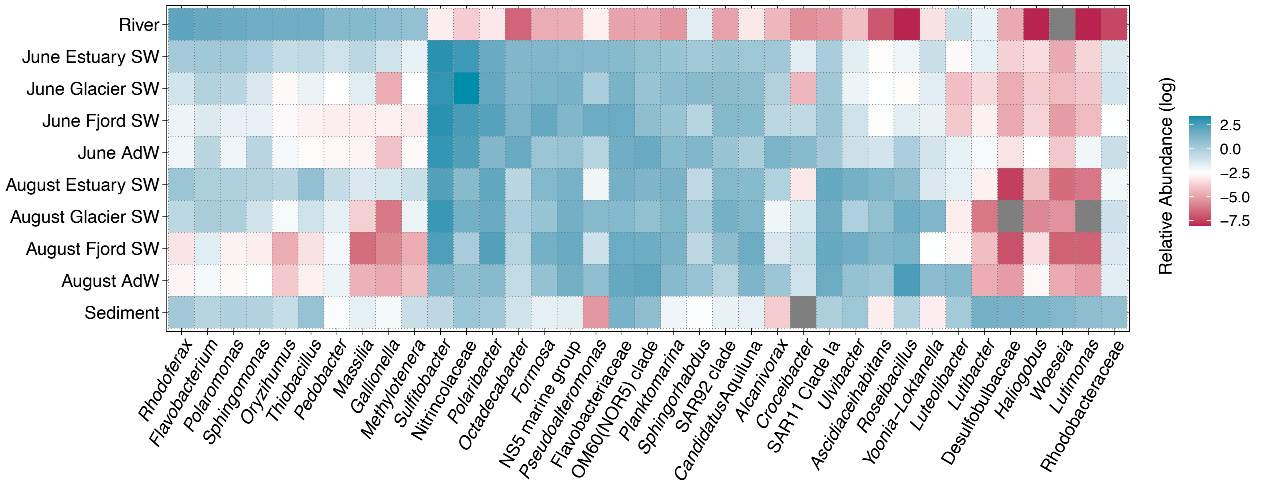

Supplementary Figure S4 | Heatmap of the mean relative abundances for the most abundant genera for each habitat and water type. Water column samples are grouped by water types. Relative abundances are shown on a log-scale for higher resolution. A high abundance is indicated by a blue color, a lower abundance is indicated by a red color, and a grey color indicates the absence of the taxon. The taxonomic affiliations of the indicators (highest specified resolution, genus or family) are indicated beneath the plot.
