# Supplementary material for: Terrestrial Inputs Shape Coastal Bacterial and Archaeal Communities in a High Arctic Fjord (Isfjorden, Svalbard)
Source: Front Microbiol. 2021 Feb 26;12:614634. doi: 10.3389/fmicb.2021.614634 (PMC7952621; doi:10.3389/fmicb.2021.614634)
Supplement: Supplementary file 5 [file Data_Sheet_5.PDF]

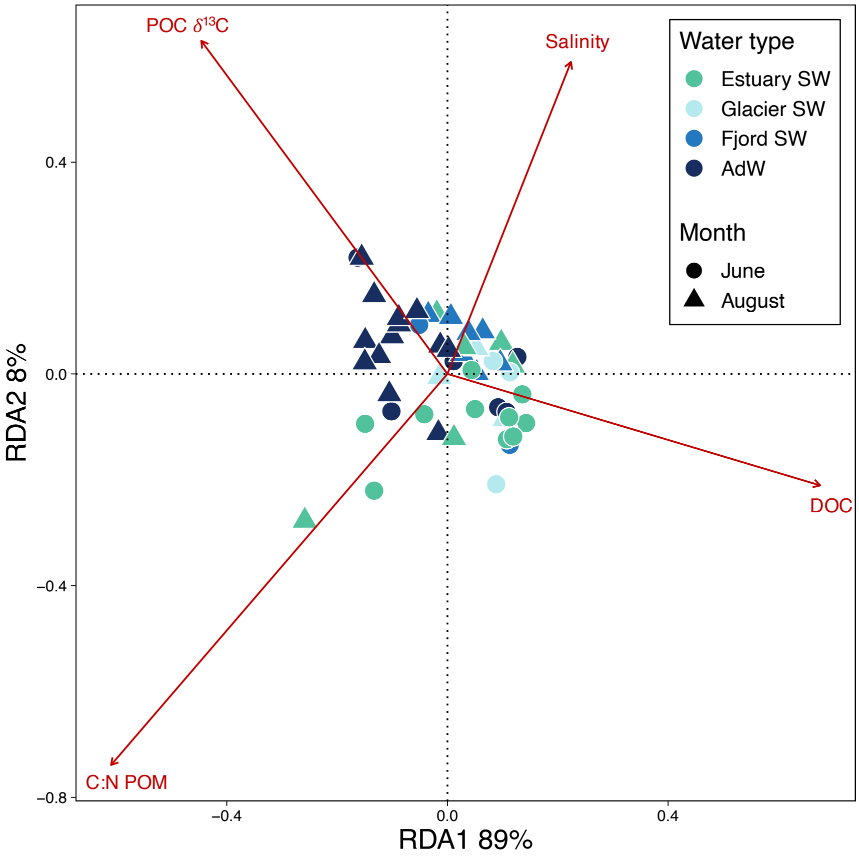

Supplementary Figure S5 | Redundancy analysis (RDA) with environmental drivers of functional traits in the fjord water column. Constraining variables are indicated in red. Percentages indicate the amount of variance explained by each axis.
