# Supplementary material for: Terrestrial Inputs Shape Coastal Bacterial and Archaeal Communities in a High Arctic Fjord (Isfjorden, Svalbard)
Source: Front Microbiol. 2021 Feb 26;12:614634. doi: 10.3389/fmicb.2021.614634 (PMC7952621; doi:10.3389/fmicb.2021.614634)
Supplement: Supplementary file 6 [file Data_Sheet_6.PDF]

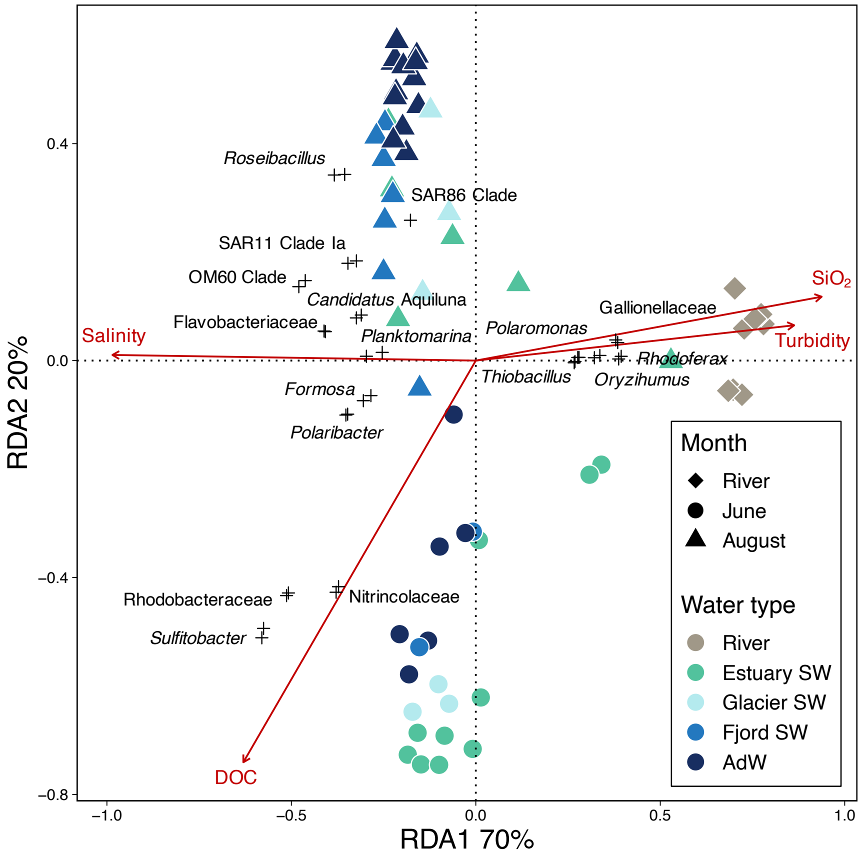

Supplementary Figure S6 | Redundancy analysis (RDA) with environmental drivers of community structure highlighting drivers of the river-fjord transition in fjord water column and river stations. Constraining variables are indicated in red. Only OTUs with an RDA score > 0.08 are represented, and are scaled by 3. The names of the closest related genus or family (highest specified taxonomic resolution) is given for these OTUs. Percentages indicate the amount of variance explained by each axis.
