# Supplementary material for: Terrestrial Inputs Shape Coastal Bacterial and Archaeal Communities in a High Arctic Fjord (Isfjorden, Svalbard)
Source: Front Microbiol. 2021 Feb 26;12:614634. doi: 10.3389/fmicb.2021.614634 (PMC7952621; doi:10.3389/fmicb.2021.614634)
Supplement: Supplementary file 7 [file Data_Sheet_7.PDF]

2 Supplementary Tables

Supplementary Table S1 | Table summing up station names, station coordinates, classification of the sample used for analysis (water type, habitat) and sampling depth. The sampling month is indicated in the sample name (Jun: June, Aug: August).

Sample name	Water type or habitat	Longitude (DD)	Latitude (DD)	Sample type	Sampling depth (m)
**A-F1_15m_Aug**	Estuary SW	78,2329	15,6912	water column	10
**A-F1_15m_Jun**	Estuary SW	78,2329	15,6912	water column	7
**A-F1_Surf_Aug**	Estuary SW	78,2329	15,6912	water column	0
**A-F1_Surf_Jun**	Estuary SW	78,2329	15,6912	water column	0
**A-F2_15m_Aug**	Estuary SW	78,2451	15,6725	water column	15
**A-F2_15m_Jun**	AdW	78,2451	15,6725	water column	15
**A-F2_Surf_Aug**	Estuary SW	78,2451	15,6725	water column	0
**A-F2_Surf_Jun**	Estuary SW	78,2451	15,6725	water column	0
**A-NC_15m_Aug**	Fjord SW	78,2712	15,5836	water column	15
**A-NC_15m_Jun**	AdW	78,2712	15,5836	water column	15
**A-NC_Surf_Aug**	Fjord SW	78,2712	15,5836	water column	0
**A-NC_Surf_Jun**	Fjord SW	78,2712	15,5836	water column	0
**Adventelva_Riv_Aug**	River	78,2044	15,8246	freshwater	0
**Adventelva_Riv_Jun**	River	78,2044	15,8246	freshwater	0
**B-Inner_15m_Aug**	AdW	78,6483	16,9037	water column	15
**B-Inner_15m_Jun**	Glacier SW	78,6483	16,9037	water column	15
**B-Inner_Surf_Aug**	Glacier SW	78,6483	16,9037	water column	0
**B-Inner_Surf_Jun**	Glacier SW	78,6483	16,9037	water column	0
**B-NC_Surf_Aug**	Fjord SW	78,588	16,5978	water column	0
**B-NC_Surf_Jun**	Fjord SW	78,588	16,5978	water column	0
**B-Outer_15m_Aug**	AdW	78,5076	16,2397	water column	15
**B-Outer_15m_Jun**	Fjord SW	78,5076	16,2397	water column	15
**B-Outer_Surf_Aug**	Fjord SW	78,5076	16,2397	water column	0
**B-Outer_Surf_Jun**	Fjord SW	78,5076	16,2397	water column	0
**B-RE_15m_Jun**	Estuary SW	78,7020	16,5838	water column	15
**B-RE_Surf_Aug**	AdW	78,7020	16,5838	water column	0
**B-RE_Surf_Jun**	Estuary SW	78,7020	16,5838	water column	0
**Bolterdalen_Riv_Aug**	River	78,1660	15,9775	freshwater	0
**Bolterdalen_Riv_Jun**	River	78,1660	15,9775	freshwater	0
**Degeerelva_Riv_Aug**	River	78,3383	16,3123	freshwater	0
**Ebbaelva_Riv_Aug**	River	78,7083	16,5997	freshwater	0
**Endalen_Riv_Aug**	River	78,1947	15,8101	freshwater	0
**Endalen_Riv_Jun**	River	78,1947	15,8101	freshwater	0
**Foxelva_Riv_Aug**	River	78,1658	16,1824	freshwater	0
**Gipselva_Riv_Aug**	River	78,4408	16,5769	freshwater	0
**ISA_15m_Aug**	AdW	78,26	15,5545	water column	15
**ISA_15m_Jun**	AdW	78,26	15,5545	water column	15
**ISA_Surf_Aug**	AdW	78,26	15,5545	water column	0
**ISA_Surf_Jun**	AdW	78,26	15,5545	water column	0
**ISG_15m_Aug**	AdW	78,129	13,999	water column	15
**ISG_15m_Jun**	AdW	78,129	13,999	water column	15
**ISG_Surf_Aug**	Fjord SW	78,129	13,999	water column	0
**ISG_Surf_Jun**	Fjord SW	78,129	13,999	water column	0
**ISK_15m_Aug**	AdW	78,3212	15,1667	water column	15
**ISK_15m_Jun**	AdW	78,3212	15,1667	water column	15

**ISK_Surf_Aug**	AdW	78,3212	15,1667	water column	0
**ISK_Surf_Jun**	Fjord SW	78,3212	15,1667	water column	0
**ME-3_15m_Aug**	AdW	78,4267	15,8263	water column	15
**ME-3_15m_Jun**	AdW	78,4267	15,8263	water column	15
**ME-3_Surf_Aug**	Fjord SW	78,4267	15,8263	water column	0
**ME-3_Surf_Jun**	Fjord SW	78,4267	15,8263	water column	0
**Sassenelva_Riv_Aug**	River	78,3320	16,8612	freshwater	0
**T-Inner_15m_Aug**	AdW	78,4335	17,2752	water column	15
**T-Inner_15m_Jun**	AdW	78,4335	17,2752	water column	15
**T-Inner_Surf_Aug**	Glacier SW	78,4335	17,2752	water column	0
**T-Inner_Surf_Jun**	Glacier SW	78,4335	17,2752	water column	0
**T-NC_15m_Aug**	AdW	78,4252	17,0871	water column	12
**T-NC_15m_Jun**	AdW	78,4252	17,0871	water column	15
**T-NC_Surf_Aug**	Glacier SW	78,4252	17,0871	water column	0
**T-NC_Surf_Jun**	Glacier SW	78,4252	17,0871	water column	0
**T-Outer_15m_Aug**	AdW	78,3785	16,4703	water column	15
**T-Outer_15m_Jun**	Fjord SW	78,3785	16,4703	water column	15
**T-Outer_Surf_Aug**	Fjord SW	78,3785	16,4703	water column	0
**T-Outer_Surf_Jun**	Fjord SW	78,3785	16,4703	water column	0
**T-RE-Degeer_15m_Aug**	AdW	78,3468	16,3625	water column	11
**T-RE-Degeer_15m_Jun**	Estuary SW	78,3468	16,3625	water column	11
**T-RE-Degeer_Surf_Aug**	Estuary SW	78,3468	16,3625	water column	0
**T-RE-Degeer_Surf_Jun**	Estuary SW	78,3468	16,3625	water column	0
**T-RE-Gips_15m_Jun**	Estuary SW	78,4358	16,5536	water column	15
**T-RE-Gips_Surf_Aug**	Estuary SW	78,4358	16,5536	water column	0
**T-RE-Gips_Surf_Jun**	Estuary SW	78,4358	16,5536	water column	0
**T-RE-Sassen_15m_Aug**	AdW	78,3482	16,8015	water column	15
**T-RE-Sassen_Surf_Aug**	Estuary SW	78,3482	16,8015	water column	0
**T-RE-Sassen_Surf_Jun**	Estuary SW	78,3482	16,8015	water column	0
**Todalen_Riv_Aug**	River	78,1779	15,8830	freshwater	0
**Todalen_Riv_Jun**	River	78,1779	15,8830	freshwater	0
**A-F1_Sed_Aug**	Sediment	78,2329	15,6912	sediment	7
**A-F2_Sed_Aug**	Sediment	78,24512	15,6725	sediment	43
**A-NC_Sed_Aug**	Sediment	78,27122	15,5836	sediment	23,3
**B-F1_Sed_Aug**	Sediment	78,652	16,516	sediment	65,6
**B-Ice_Sed_Aug**	Sediment	78,5395	16,3493	sediment	86,3
**B-Inner_Sed_Aug**	Sediment	78,6483	16,9037	sediment	46,3
**B-Inner2_Sed-Aug**	Sediment	78,652	16,937	sediment	26,6
**B-Inner3_Sed-aug**	Sediment	78,6607	16,9017	sediment	31,5
**B-Outer2_Sed_Aug**	Sediment	78,4862	16,3313	sediment	39,7
**B-Outer3_Sed-Aug**	Sediment	78,4747	16,1092	sediment	87,5
**ME-3_Sed_Aug**	Sediment	78,4267	15,8263	sediment	213,4
**T-F1_Sed_Aug**	Sediment	78,4048	17,0558	sediment	83,5
**T-Ice_Sed_Aug**	Sediment	78,3713	16,8658	sediment	98
**T-Inner_Sed_Aug**	Sediment	78,4335	17,2752	sediment	41,5
**T-NC_Sed_Aug**	Sediment	78,4252	17,0871	sediment	15,7
**T-Outer_Sed_Aug**	Sediment	78,3785	16,4703	sediment	42,7
**T-RE-Degeer_Sed_Aug**	Sediment	78,3468	16,3625	sediment	16
**T-RE-Gips_Sed_Aug**	Sediment	78,4358	16,5536	sediment	10
**T-RE-Sassen_Sed_Aug**	Sediment	78,3482	16,8015	sediment	7
