# Supplementary material for: Terrestrial Inputs Shape Coastal Bacterial and Archaeal Communities in a High Arctic Fjord (Isfjorden, Svalbard)
Source: Front Microbiol. 2021 Feb 26;12:614634. doi: 10.3389/fmicb.2021.614634 (PMC7952621; doi:10.3389/fmicb.2021.614634)
Supplement: Supplementary file 8 [file Data_Sheet_8.PDF]

Supplementary Table S2 | Table detailing KOs used for the prediction of targeted metabolic functions.

Function	KEGG Pathway	KEGG Enzyme	KEGG Genes	KEGG Orthologs (KO)
**Carbon fixation by prokaryotic organisms**	map00720	-	-	-
**Carbon fixation by photosynthetic organisms**	map00710	-	-	-
**Photosynthesis**	map00195	-	-	-
**Denitrification**	-	EC :1.7.99.4<br>EC :1.9.6.1<br>EC :1.7.2.1<br>EC :1.7.2.5<br>EC :1.7.2.4	*narGHI*<br>*napAB*<br>*nirKS*<br>*norBC*<br>*nosZ*	K0370-74<br>K02567-68<br>K15864/K00368<br>K04561/K02305<br>K00376
**Nitrification**	-	EC :1.4.99.39<br>EC :1.7.3.4	*amoCAB*<br>*hao*	K10944-46<br>K10535
**Nitrogen fixation**	-	EC :1.18.6.1	*nifDKH*<br>*anfG*	K00531<br>K02586<br>K02588<br>K02591
**Phosphate utilization (oganic)**	-	EC:3.6.1.11	*ppx*	K01524
**Sulfate reduction (dissimilatory and assimilatory)**	-	EC:2.7.7.4<br>EC:2.7.7.4<br>EC:2.7.1.25<br>EC:2.7.1.25<br>EC:1.8.4.8<br>EC :1.8.1.2<br>EC :1.8.7.1<br>EC :1.8.99.2<br>EC :1.8.99.3	*sat*<br>*cysND*<br>*PAPSS*<br>*cysC*<br>*cysH*<br>*cysπ*<br>*sir*<br>*aprAB*<br>*dsrAB*	K00958<br>K00956-57<br><br>K00860<br>K00390<br>K00380-81<br>K00392<br>K00394-95<br>K11180-81
**Methanogenesis**	-	EC :1.2.99.5<br>EC :2.3.1.101<br>EC :1.5.99.9<br>EC :1.2.98.2<br>EC :2.1.1.86<br>EC :2.8.4.1<br>EC :1.8.98.1<br>EC :1.8.98.1	*fwdABCDEFGH*<br>*ftr*<br>*mtd*<br>*hmd*<br>*mtrABCDEFGH*<br>*mcrABG*<br>*hdrA2B2C2*<br>*hdrD*	K00200-05<br>K00672<br>K00319<br>K13942<br>K00577-84<br>K00399/K00401-02<br>K03388-90<br>K08264
**Methane oxidation**	-	EC :1.1.2.7<br>EC :1.14.13.25<br>EC :1.1.2.7	Mdh12<br>mmoXYZBCD<br>mxaJGACKLD	K14028-29<br>K16157-61<br>K16254-60
