# Supplementary material for: Terrestrial Inputs Shape Coastal Bacterial and Archaeal Communities in a High Arctic Fjord (Isfjorden, Svalbard)
Source: Front Microbiol. 2021 Feb 26;12:614634. doi: 10.3389/fmicb.2021.614634 (PMC7952621; doi:10.3389/fmicb.2021.614634)
Supplement: Supplementary file 9 [file Data_Sheet_9.PDF]

Supplementary Table S3 | Table providing metadata about number of reads per sample at each step of the analysis. The column **Raw** refers to the number of paired-end reads before any bioinformatic processing. **NA** indicates that the sample was removed for further analysis. The percentage of reads retained was calculated relative to the unrarefied dataset (column **Control removed**).

Sample name	Raw	After bioinformatic processing	Mitochondria & chloroplasts removed	Singletons removed	Control removed	Rarefied	% reads retained
**A.F1_15m_Aug**	6907	6421	5816	5617	5617	**NA**	**NA**
A.F1_15m_Jun	366207	339939	206724	181690	181662	42990	24 %
A.F1_Sed_Aug	56561	52675	51306	47266	47266	42990	91 %
A.F1_Surf_Aug	85706	80426	62090	60407	60396	42990	71 %
A.F1_Surf_Jun	363213	337309	284410	207787	207782	42990	21 %
A.F2_15m_Aug	401890	374269	343223	321463	321378	42990	13 %
A.F2_15m_Jun	311069	292945	77748	74194	74163	42990	58 %
A.F2_Sed_Aug	136697	126632	123365	110608	110607	42990	39 %
A.F2_Surf_Aug	123590	116171	93337	91265	91239	42990	47 %
A.F2_Surf_Jun	74670	69210	54298	46505	46504	42990	92 %
A.NC_15m_Aug	160927	150837	130645	125692	125391	42990	34 %
A.NC_15m_Jun	117848	109928	80839	76378	76270	42990	56 %
A.NC_Sed_Aug	280815	262491	249987	203630	203629	42990	21 %
A.NC_Surf_Aug	204303	189094	174297	167519	167491	42990	26 %
A.NC_Surf_Jun	226883	211546	104655	91375	91370	42990	47 %
Adventelva_Riv_Aug	143190	132823	122779	111740	111538	42990	39 %
**Adventelva_Riv_Jun**	726	668	649	540	540	**NA**	**NA**
B.F1_Sed_Aug	110216	101507	99273	82638	82637	42990	52 %
**B.Ice_Sed_Aug**	28163	25937	24641	20688	20687	**NA**	**NA**
B.Inner2_Sed_Aug	165967	124166	118271	109459	109456	42990	39 %
B.Inner3_Sed_Aug	567706	143674	139336	124275	124274	42990	35 %
B.Inner_15m_Aug	167016	155186	150827	146404	146345	42990	29 %
B.Inner_15m_Jun	375529	525700	471537	439458	439335	42990	10 %
B.Inner_Sed_Aug	211034	155232	152094	136496	136492	42990	31 %
B.Inner_Surf_Aug	133734	350495	265219	227610	227608	42990	19 %
B.Inner_Surf_Jun	155213	197717	164609	160251	160248	42990	27 %
B.NC_Surf_Aug	162432	151430	145316	141239	141237	42990	30 %
B.NC_Surf_Jun	276845	258553	234860	228141	228139	42990	19 %
B.Outer2_Sed_Aug	6309	228216	222187	166635	166635	42990	26 %
B.Outer3_Sed_Aug	112594	202550	197397	165134	165134	42990	26 %
**B.Outer_15m_Aug**	312932	5921	4361	4331	4331	**NA**	**NA**
B.Outer_15m_Jun	188915	104857	97679	95770	95764	42990	45 %
B.Outer_Surf_Aug	246925	291074	250511	243278	243100	42990	18 %
B.Outer_Surf_Jun	219427	176410	173111	169491	169482	42990	25 %
B.RE_15m_Jun	152857	142661	122369	119738	119705	42990	36 %
B.RE_Surf_Aug	266500	248142	243047	234763	234743	42990	18 %
B.RE_Surf_Jun	81492	76220	65898	63245	63243	42990	68 %
Bolterdalen_Riv_Aug	357622	327804	300390	264340	264330	42990	16 %
Bolterdalen_Riv_Jun	67062	61722	52854	42990	42990	42990	100 %
**Control_PCR_Seq**	817	746	746	741	**NA**	**NA**	**NA**
Degeerelva_Riv_Aug	295677	271189	262482	229198	229085	42990	19 %
Ebbaelva_Riv_Aug	116327	107472	88652	76965	76949	42990	56 %
**Endalen_Riv_Aug**	3640	3340	3088	2860	2859	**NA**	**NA**
Endalen_Riv_Jun	132617	122717	116935	97605	97603	42990	44 %
Foxelva_Riv_Aug	143226	132185	120558	109301	109300	42990	39 %
Gipselva_Riv_Aug	145525	134400	116063	84363	84250	42990	51 %
ISA_15m_Aug	214121	199439	192981	185437	185109	42990	23 %
ISA_15m_Jun	329662	306545	237716	222619	222502	42990	19 %
ISA_Surf_Aug	255090	237048	222100	204374	204049	42990	21 %
ISA_Surf_Jun	203359	188794	146140	129884	129842	42990	33 %

ISG_15m_Aug	90181	84677	76316	72452	72404	42990	59 %
ISG_15m_Jun	518111	482219	268109	259525	259478	42990	17 %
ISG_Surf_Aug	143909	134822	127904	122088	121991	42990	35 %
ISG_Surf_Jun	221987	207520	187823	179142	179142	42990	24 %
ISK_15m_Aug	222888	207264	196377	188705	188597	42990	23 %
ISK_15m_Jun	80436	75839	50293	49070	49058	42990	88 %
ISK_Surf_Aug	69334	64573	61224	59635	59299	42990	72 %
ISK_Surf_Jun	305268	283811	193380	188514	188512	42990	23 %
ME.3_15m_Aug	353641	328366	301521	291863	291607	42990	15 %
ME.3_15m_Jun	254177	237596	211275	206908	206902	42990	21 %
ME.3_Sed_Aug	157949	146442	123378	102696	102696	42990	42 %
ME.3_Surf_Aug	324216	303375	287387	275654	275628	42990	16 %
ME.3_Surf_Jun	640368	598748	450080	421098	421090	42990	10 %
Sassenelva_Riv_Aug	260250	239065	222810	168607	168480	42990	26 %
T.F1_Sed_Aug	176267	163709	160080	144449	144449	42990	30 %
T.Ice_Sed_Aug	124851	115391	111479	99829	99829	42990	43 %
T.Inner_15m_Aug	250678	233863	226956	217534	217491	42990	20 %
T.Inner_15m_Jun	164103	154333	96206	93499	93495	42990	46 %
T.Inner_Sed_Aug	219686	202534	190862	178956	178512	42990	24 %
T.Inner_Surf_Aug	102370	95483	58037	56245	56221	42990	76 %
T.Inner_Surf_Jun	219919	205060	124246	118958	118949	42990	36 %
T.NC_15m_Aug	167544	156698	145785	140554	140528	42990	31 %
T.NC_15m_Jun	464712	436183	225922	214819	214679	42990	20 %
**T.NC_Sed_Aug**	904	842	781	682	681	**NA**	**NA**
T.NC_Surf_Aug	184353	172192	134864	130871	130724	42990	33 %
**T.NC_Surf_Jun**	1093	1007	696	673	673	**NA**	**NA**
T.Outer_15m_Aug	117994	110467	108446	105937	105620	42990	41 %
T.Outer_15m_Jun	151298	140881	139916	137312	137303	42990	31 %
T.Outer_Sed_Aug	168142	155604	151117	112201	112201	42990	38 %
T.Outer_Surf_Aug	180694	168905	164465	159510	159433	42990	27 %
T.Outer_Surf_Jun	328948	303825	258523	247064	246938	42990	17 %
T.RE.Degeer_15m_Aug	140449	131295	122010	117951	117915	42990	36 %
T.RE.Degeer_15m_Jun	435739	405301	289680	270410	270332	42990	16 %
T.RE.Degeer_Sed_Aug	130037	119945	112145	95252	95250	42990	45 %
T.RE.Degeer_Surf_Aug	112068	104492	76957	71467	71463	42990	60 %
T.RE.Degeer_Surf_Jun	269496	252601	146834	137427	137417	42990	31 %
T.RE.Gips_15m_Jun	130834	122696	47767	46249	46216	42990	93 %
T.RE.Gips_Sed_Aug	221399	206638	196500	171059	170964	42990	25 %
T.RE.Gips_Surf_Aug	126981	118667	82064	76228	76212	42990	56 %
T.RE.Gips_Surf_Jun	344357	323642	242823	226548	226537	42990	19 %
T.RE.Sassen_15m_Aug	247476	231966	218992	198095	198073	42990	22 %
**T.RE.Sassen_Sed_Aug**	25769	23755	22616	20403	20402	**NA**	**NA**
T.RE.Sassen_Surf_Aug	334786	309378	282026	249274	248991	42990	17 %
T.RE.Sassen_Surf_Jun	294625	273817	223367	203449	203446	42990	21 %
Todalen_Riv_Aug	182669	168799	159139	145059	145051	42990	30 %
Todalen_Riv_Jun	367493	338283	307270	215416	215416	42990	20 %
**Total number reads**	19298202	17955002	14955864	13642734	13636601	3740130	27 %
**Total number OTUs**	-	1000646	863136	35576	35576	35280	99 %
