# Supplementary material for: Terrestrial Inputs Shape Coastal Bacterial and Archaeal Communities in a High Arctic Fjord (Isfjorden, Svalbard)
Source: Front Microbiol. 2021 Feb 26;12:614634. doi: 10.3389/fmicb.2021.614634 (PMC7952621; doi:10.3389/fmicb.2021.614634)
Supplement: Supplementary file 10 [file Data_Sheet_10.PDF]

Supplementary Table S4 | Statistical outputs for pairwise comparisons of alpha diversity indices giving FDR-corrected *p*-values for Dunn's *post hoc* test performed on a Kruskal-Wallis test to account for non-gaussian repartitions. (**A**) Comparisons of groups separating habitat and month in the water column. (**B**) Comparisons of groups separating water type. These tests correspond to the ones represented on the **Supplementary Figure S1** and **S2**. Significant *p*-values are written in black.

A

Comparisons	nOTU	Chao1	ACE	Pielou's evenness	Shannon	Inverse Simpson's	Relative singletons
August - June	$1,58.10^{-02}$	$1,55.10^{-03}$	$1,77.10^{-03}$	$6,81.10^{-04}$	$9,66.10^{-03}$	$8,20.10^{-04}$	$2,00.10^{-03}$
August - River	$7,14.10^{-06}$	$3,05.10^{-04}$	$5,98.10^{-04}$	$4,82.10^{-04}$	$1,38.10^{-04}$	$5,19.10^{-04}$	$5,10.10^{-04}$
June - River	$2,15.10^{-03}$	$1,00.10^{-01}$	$1,31.10^{-01}$	$6,42.10^{-09}$	$3,48.10^{-08}$	$9,16.10^{-09}$	$2,77.10^{-08}$
August - Sediment	$2,38.10^{-07}$	$9,16.10^{-07}$	$2,35.10^{-06}$	$3,20.10^{-05}$	$8,49.10^{-06}$	$1,13.10^{-05}$	$1,30.10^{-03}$
June - Sediment	$3,61.10^{-04}$	$9,52.10^{-03}$	$1,45.10^{-02}$	$8,37.10^{-12}$	$1,35.10^{-10}$	$2,12.10^{-12}$	$2,35.10^{-08}$
River - Sediment	$4,47.10^{-01}$	$2,44.10^{-01}$	$2,39.10^{-01}$	$4,23.10^{-01}$	$4,48.10^{-01}$	$3,43.10^{-01}$	$2,83.10^{-01}$

B

Comparisons	nOTU	Chao1	ACE	Pielou's evenness	Shannon	Inverse Simpson's	Relative singletons
AdW - Estuary SW	$2,24.10^{-02}$	$8,02.10^{-03}$	$4,65.10^{-03}$	$4,93.10^{-01}$	$3,26.10^{-01}$	$4,99.10^{-01}$	$6,90.10^{-02}$
AdW - Fjord SW	$3,11.10^{-01}$	$3,22.10^{-01}$	$3,49.10^{-01}$	$1,06.10^{-01}$	$7,83.10^{-02}$	$1,62.10^{-01}$	$2,15.10^{-01}$
Estuary SW - Fjord SW	$1,01.10^{-02}$	$3,83.10^{-03}$	$2,73.10^{-03}$	$1,20.10^{-01}$	$4,13.10^{-02}$	$1,80.10^{-01}$	$2,60.10^{-01}$
AdW - Glacier SW	$3,95.10^{-01}$	$3,17.10^{-01}$	$2,90.10^{-01}$	$1,94.10^{-01}$	$2,03.10^{-01}$	$1,77.10^{-01}$	$2,64.10^{-01}$
Estuary SW - Glacier SW	$1,31.10^{-01}$	$1,16.10^{-01}$	$1,05.10^{-01}$	$2,00.10^{-01}$	$1,34.10^{-01}$	$1,86.10^{-01}$	$3,41.10^{-01}$
Fjord SW - Glacier SW	$2,76.10^{-01}$	$2,19.10^{-01}$	$2,12.10^{-01}$	$4,89.10^{-01}$	$4,31.10^{-01}$	$4,15.10^{-01}$	$4,74.10^{-01}$
AdW - River	$3,50.10^{-05}$	$3,08.10^{-03}$	$4,07.10^{-03}$	$4,37.10^{-05}$	$3,15.10^{-05}$	$4,20.10^{-05}$	$1,77.10^{-04}$
Estuary SW - River	$1,94.10^{-02}$	$2,87.10^{-01}$	$3,77.10^{-01}$	$1,14.10^{-04}$	$3,45.10^{-04}$	$1,04.10^{-04}$	$2,00.10^{-06}$
Fjord SW - River	$1,56.10^{-05}$	$1,49.10^{-03}$	$2,39.10^{-03}$	$1,04.10^{-06}$	$3,52.10^{-07}$	$2,86.10^{-06}$	$2,78.10^{-05}$
Glacier SW - River	$4,01.10^{-03}$	$5,94.10^{-02}$	$7,74.10^{-02}$	$1,38.10^{-04}$	$1,24.10^{-04}$	$1,06.10^{-04}$	$7,56.10^{-04}$
AdW - Sediment	$1,86.10^{-06}$	$4,68.10^{-05}$	$6,65.10^{-05}$	$1,74.10^{-06}$	$1,63.10^{-06}$	$4,76.10^{-07}$	$4,35.10^{-04}$
Estuary SW - Sediment	$7,43.10^{-03}$	$8,19.10^{-02}$	$1,28.10^{-01}$	$8,65.10^{-06}$	$4,71.10^{-05}$	$2,61.10^{-06}$	$3,87.10^{-06}$
Fjord SW - Sediment	$9,72.10^{-07}$	$2,46.10^{-05}$	$4,57.10^{-05}$	$2,21.10^{-08}$	$8,46.10^{-09}$	$2,21.10^{-08}$	$6,45.10^{-05}$
Glacier SW - Sediment	$1,76.10^{-03}$	$1,31.10^{-02}$	$1,83.10^{-02}$	$2,98.10^{-05}$	$3,27.10^{-05}$	$1,03.10^{-05}$	$1,91.10^{-03}$
River - Sediment	$4,47.10^{-01}$	$2,44.10^{-01}$	$2,39.10^{-01}$	$4,23.10^{-01}$	$4,48.10^{-01}$	$3,43.10^{-01}$	$2,83.10^{-01}$
