# Supplementary material for: Terrestrial Inputs Shape Coastal Bacterial and Archaeal Communities in a High Arctic Fjord (Isfjorden, Svalbard)
Source: Front Microbiol. 2021 Feb 26;12:614634. doi: 10.3389/fmicb.2021.614634 (PMC7952621; doi:10.3389/fmicb.2021.614634)
Supplement: Supplementary file 11 [file Data_Sheet_11.PDF]

Supplementary Table S5 | Mean relative abundances (%) of the most abundant classes by month and water type. This table summarizes **Figure 4A** in a simplified way.

	**River**	**June**				**August**				**Sediment**
		Estuary SW	Glacier SW	Fjord SW	AdW	Estuary SW	Glacier SW	Fjord SW	AdW	
Gammaproteobacteria	41.3	37.6	42.9	34.5	33.5	26.1	25.2	18.6	26.1	35.3
Alphaproteobacteria	11.8	33.0	31.1	31.3	36.8	27.7	37.4	27.9	22.1	9.3
Bacteroidia	17.1	20.9	21.3	28.1	19.6	32.3	24.2	39.2	24.8	25.5
Actinobacteria	11.1	3.7	3.3	4.3	4.7	4.6	4.3	7.1	7.1	1.3
Verrucomicrobiae	1.9	0.8	0.2	0.5	2.4	3.0	5.4	4.1	15.1	4.0
Campylobacteria	2.0	0.2	0.1	0.1	0.2	1.2	0.3	0.1	0.1	2.1
Gemmatimonadetes	3.2	0.9	0.2	0.1	0.1	0.8	0.3	0.0	0.0	0.9
Acidimicrobiia	1.3	0.9	0.5	0.7	1.9	1.8	2.5	2.6	3.8	1.7
Nitrososphaeria	0	0	0	0	0	0	0	0	0	1.1
Thermoanaerobaculia	0	0	0	0	0	0	0	0	0	1.3
Desulfobulbia	0.1	0.1	0	0	0.1	0	0	0	0	6.3
Desulfobacteria	0	0	0	0	0	0	0	0	0	1.8
Anaerolineae	0.2	0	0	0	0	0.1	0	0	0	1.1
Thermoleophilia	2.0	0.4	0.1	0.1	0.1	0.5	0.1	0	0	0.2
Bacilli	0.1	0	0	0.1	0	0.1	0	0	0.1	1.0
