# Supplementary material for: Terrestrial Inputs Shape Coastal Bacterial and Archaeal Communities in a High Arctic Fjord (Isfjorden, Svalbard)
Source: Front Microbiol. 2021 Feb 26;12:614634. doi: 10.3389/fmicb.2021.614634 (PMC7952621; doi:10.3389/fmicb.2021.614634)
Supplement: Supplementary file 12 [file Data_Sheet_12.PDF]

Supplementary Table S6 | Highly abundant indicators of habitats and sampling months in the water column, and number of significant OTUs making up each taxon. Indicators were filtered with an Indicator Value ≥ 0.7, a *p*-value ≤ 0.001 and a minimum relative abundance threshold of 0.5% within their group. The permutation number was set to 9999 using the function *how* in the permute package in R. Indicators are ordered by decreasing abundance.

Group	Indicator	Number of OTUs
August	*Sulfitobacter*	3
	Roseibacillus	15
	SAR 11 Clade Ia	12
	Polaribacter	4
	OM60(NOR5) clade	5
	Flavobacteriaceae	13
	NS5 marine group	22
	Candidatus Aquiluna	4
	Planktomarina	9
	Ulvibacter	6
	Ascidiaceihabitans	6
	SAR92 clade	4
	Cryomorphaceae	12
	Yoonia-Loktanella	2
	SUP05 cluster	2
	Fluviicola	6
	Sva0996 marine group	2
	Ilumatobacter	4
	Luteolibacter	3
	Halomonas	2
	OM43 clade	2
	Saprospiraceae	3
	Pseudohongiella	4
	Hyphomonas	3
	Hoeflea	2
	NS4 marine group	6
	Psychrobacter	6
	Zhongshania	2
June	*Sulfitobacter*	19
	Nitrincolaceae	29
	Polaribacter	17
	Flavobacteriaceae	13
	Octadecabacter	6
	Formosa	8
	OM60(NOR5) clade	1
	NS5 marine group	8
	Pseudoalteromonas	3
	SAR92 clade	11
	Planktomarina	1
	Sphingorhabdus	2
	Alcanivorax	2
	Methylophagaceae	4
	OM43 clade	1
	Granulosicoccus	5
River	*Rhodoferax*	11
	Flavobacterium	70
	Polaromonas	7
	Sphingomonas	50
	Oryzihumus	16
	Thiobacillus	9
	Pedobacter	31
	Massilia	18
	Gallionella	17
	Methylotenera	11
	Ferruginibacter	30
	Sulfuricurvum	3
	Gemmatimonadaceae	31

	Arenimonas	10
	Novosphingobium	12
	hgcI clade	11
	Gemmatimonas	32
	Gaiella	20
	Parafrigoribacterium	1
	Rhizobacter	3
	Ellin6067	9
	Sulfuriferula	10
	Mucilaginibacter	21
	Bdellovibrio	29
	Rhodanobacter	8
	CL500-29 marine group	12
	Chitinophagaceae	9
	Cryobacterium	2
	Brevundimonas	4
	Bacteriovorax	13
	Algoriphagus	2
	Haliangium	13
	Nocardioides	11
	Legionella	15
	Alkanindiges	5
**Sediment**	Desulfobulbaceae	24
	Lutibacter	17
	Halioglobus	34
	Flavobacteriaceae	67
	Woeseia	35
	Lutimonas	12
	OM60(NOR5) clade	10
	Rhodobacteraceae	55
	Nitrincolaceae	10
	Ulvibacter	8
	Cyclobacteriaceae	55
	Colwellia	15
	Psychromonas	9
	Sandaracinaceae	6
	Saprospiraceae	44
	Luteolibacter	3
	Polaribacter	2
	Candidatus Nitrosopumilus	9
	Anaerolineaceae	14
	Subgroup 10	25
	Sva0996 marine group	20
	Limibaculum	5
	Methylophagaceae	3
	R76-B128	29
	Arcobacteraceae	5
	Persicirhabdus	8
	Roseibacillus	13
	Aquibacter	11
	Desulforhopalus	7
	Sulfurovum	6
	SEEP-SRB1	2
	SEEP-SRB4	4
	Gemmatimonadaceae	9
	Izimaplasma	8
	Sva0081 sediment group	7
	C1-B045	3
	Vicingus	8
	BD1-7 clade	26
